# Strategic Innovation Trends in Small and Medium Industry Clusters for Enhancing Regional Tourism Competitiveness: A Bibliometric Analysis

**DOI:** 10.12688/f1000research.183398.1

**Published:** 2026-06-24

**Authors:** Christian Kuswibowo, Siti Jahroh, Alim Setiawan Slamet, Harianto Harianto

**Affiliations:** 1 IPB University, School of Business, Bogor, Indonesia; 2 Marketing Management Study Program, Polytechnic APP Jakarta, Jakarta, Indonesia; 3 IPB University, Faculty of Economics and Management, Bogor, Indonesia

**Keywords:** Strategic Innovation, Small and Medium Industries, Regional Tourism, Competitiveness, Sustainability, Bibliometric Analysis

## Abstract

**Objective:**

This bibliometric study systematically evaluates the multi-dimensional evolution of strategic innovation trends within Small and Medium Industry (SMI) clusters between 2005 and 2025. It synthesizes the structural interplay between internal digitalization capabilities, collaborative networking, and cluster-based regional configurations, mapping how these interconnected strategic factors act as overriding drivers to enhance objective regional tourism competitiveness and long-term economic sustainability.

**Methods:**

A rigorous bibliometric mapping and systematic review were performed using custom-built PRISMA 2020 and PICOC frameworks across the Scopus and Web of Science global databases. From an initial identification pool of 3,295 records, a strict screening process based on clear inclusion criteria (peer-reviewed final journal articles, English language, and open-access/hybrid gold status) was executed. Exactly 195 high-quality studies were selected for final visual network analysis and qualitative thematic synthesis using VOSviewer and RStudio Biblioshiny.

**Results:**

The analysis reveals that the literature has experienced a substantial expansion, boasting a 15.86% compound annual growth rate heavily driven by intensive global research collaboration. Structural co-authorship and citation mapping identify key foundational intellectual hubs such as Zhang J., Buhalis D., and Universitas Brawijaya deeply anchoring the discipline. Bibliographic coupling and keyword co-occurrence matrix networks uncover a massive thematic transformation: consumer engagement and local economic positioning are easily distorted without digital capacity building. At the structural level, a clear paradigm shift shows that traditional product/managerial innovations are now overridden by smart tourism deployment, green circular economy ecosystems, and cross-sectoral co-creation models.

**Conclusion:**

Regional tourism optimization operates within a complex socio-ecological matrix where localized industrial capabilities continuously negotiate with macro-level digital and market disruptions. While separate clusters of innovation exist, there is a critical need for integrated regional public policy frameworks that combine physical cluster infrastructure modifications with tailored digital behavioral support to achieve true long-term competitive advantages and planetary sustainability goals.

## Introduction

Small and Medium Industries (SMIs) are the backbone of the local economy in many regions of Indonesia, significantly contributing to the development of the tourism sector. Strategic innovation in SMI centres is key to increasing regional tourism competitiveness amid increasingly fierce global competition. ^1^ ^,^ ^2^ Small and Medium Industries (SMIs) serve as the backbone of the local economy across various regions in Indonesia. The existence of SMIs not only contributes to job creation and regional economic strengthening but also serves as a vital pillar in developing an increasingly competitive tourism sector in the era of globalization. In the face of rapidly changing market dynamics and fierce competition, strategic innovation emerged as a key factor that encouraged the increase of regional tourism competitiveness through strengthening SMI centres. As explained by, ^3^ the concept of strategic innovation emphasizes significant changes in products, processes, and business models that must be managed effectively for organizations, including SMIs, to survive and thrive amid external challenges.

Digitalization and technological transformation are the main drivers in the development of SMI innovations, as outlined by ^4^ ^,^ ^5^ which underscores the need for digital capacity building and cross-sector collaboration to strengthen local economic competitiveness. In the context of tourism ^5^ ^,^ ^6^ highlighting the role of digital technology in creating smart tourism destinations that improve the tourist experience and the efficiency of destination management. This confirms that strategic innovations that integrate digitalisation and sustainability aspects are very relevant to be developed in SMI centres that support regional tourism.

Comprehensive review by, ^7^ reinforcing the importance of integrating product and process innovation in the tourism sector to support sustainable growth. A collaborative approach to innovation, as put forward by, ^8^ through co-innovation, emphasises that synergy between business actors, the government, and local communities is essential to create shared value and accelerate innovation adoption. National Competitiveness Framework. ^9^ ^–^ ^14^ It also provides a strong theoretical foundation in understanding how strategic innovation can create a sustainable competitive advantage for SMIs and regional tourism destinations. In addition, global trends that increasingly prioritize aspects of sustainability and the circular economy, as reviewed by ^14^ ^–^ ^16^ is an important concern in the development of innovations that are not only oriented to economic growth but also to environmental preservation and social welfare. Social media and digital technology also significantly improve customer engagement and tourist experience, which is a vital aspect in the marketing and development of community-based tourism products and SMIs. ^17^

However, innovation management is not free from challenges, especially in overcoming organisational rigidity that can hinder the adaptation and development of new products. ^18^ Therefore, SMI centres need to adopt adaptive and sustainable innovation strategies to maintain relevance and competitiveness in a dynamic market. Based on the theoretical foundation and previous research findings, this study aims to systematically examine the development of strategic innovation trends in SMI centres that contribute to increasing regional tourism competitiveness in Indonesia. The bibliometric approach was chosen as the. An appropriate method to map the publication patterns, key actors, dominant research themes, and collaborative networks developed over the past two decades. Thus, this study is expected to provide comprehensive insights and strategic recommendations that apply to the development of innovations in the SMI and regional tourism sectors, while answering the needs of digital transformation and sustainability, which are the main demands in the modern era.

Although many studies have addressed innovation and tourism separately, the integration of the two with a systematic approach is still limited. The bibliometric approach offers a quantitative method to map research progress, identify key actors, and uncover collaboration patterns and dominant research themes. Therefore, this study aims to examine the development of strategic innovation trends related to regional tourism competitiveness in SMI centres through a bibliometric analysis of scientific publications over the past two decades. This study is expected to provide comprehensive insights and strategic recommendations for developing innovations in the SMI and tourism sectors. Therefore, based on the above explanation, several research questions were developed to guide this analysis more directly and in-depth, with some questions developed as follows.

## Materials and methods

The research methodology used in this study is categorised as descriptive. The authors used bibliographic techniques to answer research questions, citing their methodological advantages over conventional qualitative reviews due to their analytical depth and accuracy. This study uses a bibliometric analysis method by collecting data from scientific publications from the Scopus database relevant to strategic innovation, SMIs, and regional tourism during the period 2005-2025, especially for journal articles that have been finalised, in English, open access and hybrid gold. Data is collected using predefined keywords through Boolean logic designed to capture relevant and comprehensive literature. *((“strategic innovation” OR “innovation strategy” OR “strategic innovation”) AND (“small and medium enterprises” OR SMIs OR “small and medium industries” OR “SMI centers”) AND (competitiveness OR “competitive advantage” OR “market competitiveness” OR “competitiveness”) AND (tourism OR tourism OR “regional tourism” OR “local tourism” OR “tourism industry”)). From the results of the data collection, 3,295 article documents were obtained. The document was then filtered back specifically for final journal articles in English, open access, and hybrid gold. As many as 195 documents were found that were ready for further analysis.*

The analysis used bibliometric software such as VOSviewer and Bibliometrix to map publication trends, keyword analysis, and collaborative networks between authors, institutions, and countries. This approach allows the identification of research patterns, key actors, and themes that develop quantitatively and visually. The entire process is carried out systematically to ensure the validity and reliability of the data and analysis. Therefore, the power of the research design is further enhanced by incorporating custom-built PRISMA and PICOC frameworks, which contribute to an optimally comprehensive and structured approach **(**as illustrated in Figure 1).

**Figure 1.**
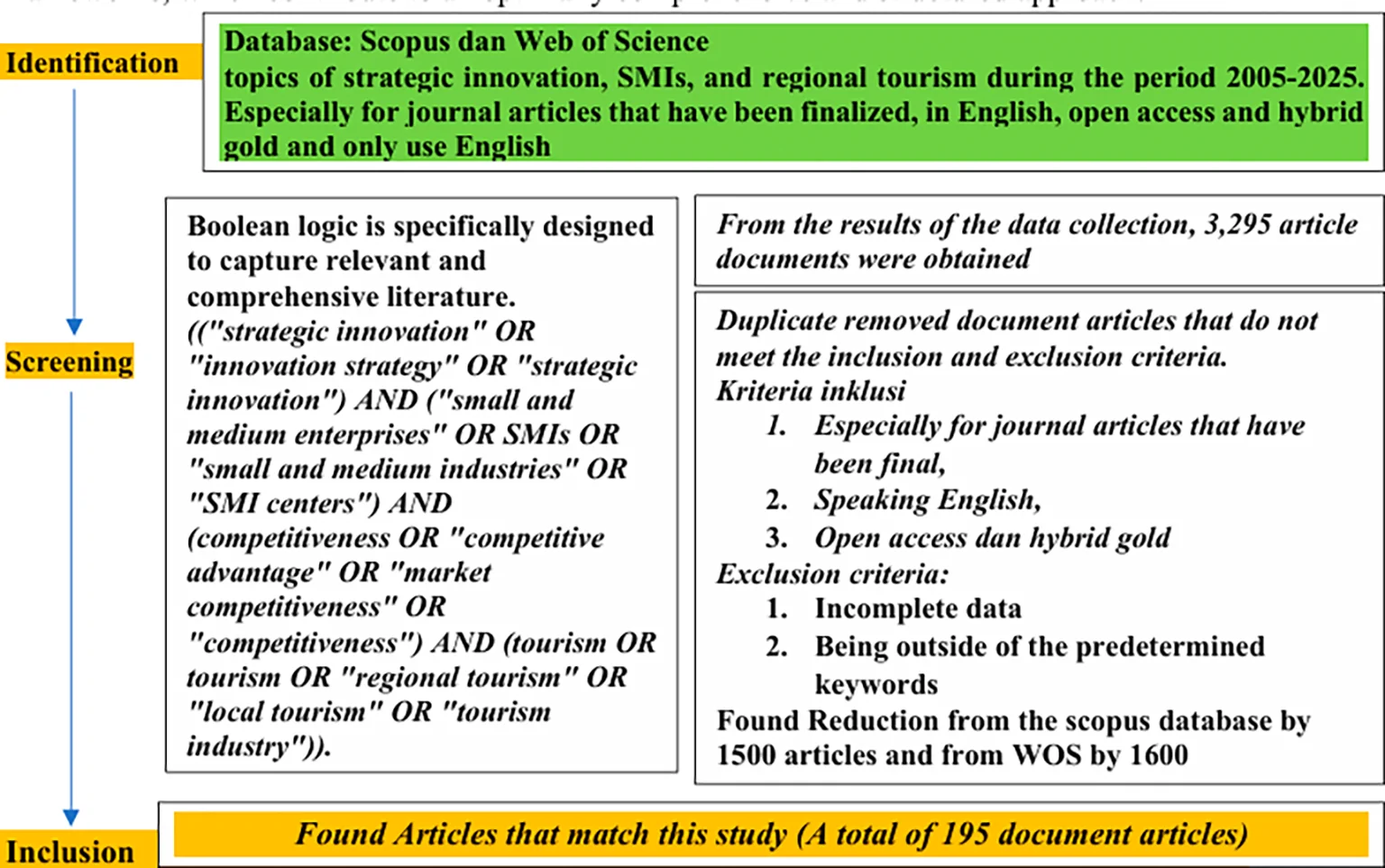
PRISMA view of the data filtering process.

The data screening process in this study follows the PRISMA ( *Preferred Reporting Items for Systematic Reviews and Meta-Analyses *) framework, which consists of three main stages: *Identification*, *Screening*, and *Inclusion.* In the Identification stage, literature searches were carried out in two leading academic databases, namely Scopus and Web of Science, with a focus on strategic innovation, Small and Medium Industries (SMIs), and regional tourism from 2005 to 2025. The data collected is limited to final journal articles in English and have open access or hybrid gold. This ensures the quality and relevance of the data sources used in the analysis.

Furthermore, at the Screening stage, Boolean logic is used, specifically designed to capture relevant and comprehensive literature. The Boolean logic combines several key terms such as “strategic innovation”, “innovation strategy”, “strategic innovation”, “small and medium enterprises”, “small and medium industries”, “SMI centers”, as well as terms related to competitiveness and tourism such as “competitiveness”, “competitive advantage”, “tourism”, and its variations. From the initial search results, 3,295 article documents were obtained. However, duplicate documents are removed, and filtering is carried out based on inclusion and exclusion criteria.

The inclusion criteria include journal articles that are final, in English, and have open access or hybrid gold status. Meanwhile, the exclusion criteria include incomplete data and articles that do not contain specific predetermined keywords. After this screening process, 1,500 articles from the Scopus database and 1,632 articles from the Web of Science were significantly reduced, leaving articles that meet quality and relevance standards. The last stage is Inclusion, where articles that pass screening and meet all criteria are included in the bibliometric analysis. At this stage, 195 documents articles were found that were within the focus of this study. These articles are the primary databases analysed to identify strategic innovation trends, key actors, dominant themes, and collaboration networks in the context of SMI centres and regional tourism competitiveness. The researcher also uses the PICOC framework as a strategic tool in formulating appropriate and focused research questions, thus allowing for a more in-depth analysis of the problems raised. The PICOC framework, which stands for Population, Intervention, *Comparison, Outcome, and Context*, serves as a guide to structure research questions by clearly defining the five components. This approach ensures that the questions asked are specific, relevant, and aligned with the study's main objectives.

In Table 1, each element of the PICOC framework is described in detail along with the resulting research questions. This structure not only helps prepare targeted questions but also organises discussions related to the main challenges of the research, so that all important aspects can be thoroughly discussed. By adopting the PICOC framework, researchers can maintain the focus and systematics of the research while increasing clarity and effectiveness in the investigation process. In the data analysis, the advanced VOSviewer software and RStudio Biblioshiny are used, with attention to various units of analysis such as authors, institutions, and countries. The analysis includes co-authorship, co-occurrence, citations, bibliographic grouping, and co-citations, with a complete count method chosen over fractional counting to ensure more comprehensive data coverage.

**Table 1.** PICOC framework specifications and research questions.

Initials	Component	Details of components	Formulation of research questions	Number RQ
P	Population	Small and Medium Industry (SMI) centers that contribute to regional tourism sectors.	How has the volume of scientific publications regarding strategic innovation in SMI centers evolved from 2005 to 2025?	RQ A (1)
I	Intervention	Implementation of strategic innovation and digital transformation within SMI centers.	How does the implementation of strategic innovation contribute to enhancing regional tourism competitiveness?	RQ E (5)
C	Comparison	Analysis of contributions and collaborative networks across different authors, institutions, and countries.	Who are the leading authors, institutions, and countries significantly contributing to this research field?	RQ B (2)
O	Outcome	Identification of dominant research themes, citation patterns, keyword trends, and practical implications.	What are the central research themes and dominant keywords that define the landscape of strategic innovation in SMIs?	RQ C (3)
C	Background	Geographical and temporal context: Regional tourism in Indonesia from 2005 to 2025	What are the citation patterns, co-citation networks, and bibliographic coupling structures within this context?	RQ D (4)

In the context of developing countries, digital transformation has become a critical element for SMI survival. As emphasized by, ^19^ the success of e-commerce adoption is heavily influenced by website quality, which serves as a foundational competitive advantage for Indonesian MSMEs in the digital marketplace. This aligns with the need for SMIs to integrate technology into their business models to enhance regional reach.

In this bibliometric analysis, visual elements such as the distance between the circles, the size of the circles, and the colour variations are used to convey commonly understood meanings, making it easier to understand the data. The spacing between the circles illustrates how strong the relationship between the units of analysis is; the size of the circle indicates the magnitude of the output or level of activity. At the same time, the colour difference marks different groups or clusters in the dataset. To address these aspects systematically, the detailed specifications of the PICOC framework along with the formulated research questions are presented in Table 1. Carefully crafted research questions are the main foothold in directing deeper discussions, ensuring that the analysis remains focused on the main objectives of the research. Carefully crafted research questions are the main foothold in directing deeper discussions, ensuring that the analysis remains focused on the main objectives of the research.

Technological innovation acts as a catalyst for sustainable growth in the modern economy. As highlighted by ^20^ in their systematic review, the integration of technology in start-ups is essential for achieving broader economic goals, such as decent work and economic growth, which are central to the resilience of industrial clusters.

To establish a solid theoretical foundation before proceeding with the bibliometric visualization, a systematic review of seminal literature was conducted. This synthesis highlights the core variables and findings from previous studies that shape the current understanding of strategic innovation in SMI clusters. The following table ( Table 2) summarizes these key contributions and serves as the conceptual basis for the analysis that follows.

**Table 2.** Matrix of research topics, innovation strategies, and regional focus across periods.

Variable/topic	Pre-pandemic (Normal)	During pandemic (Crisis)	Post-pandemic (Recovery/Future)
Regional Tourism	Focus on destination branding & infrastructure. ^21^	Focus on local safety & health protocols; travel collapse.	Sustainable, resilient, & smart tourism. ^22^
SMI Clusters	Industrial efficiency & labor-based clusters. ^1^	Supply chain vulnerability; shift to virtual collaboration.	Innovation-driven clusters & knowledge dynamics. ^13^
Strategic Innovation	Traditional product & managerial innovation. ^3^	Survival-based digitalization & business model shifts.	Green innovation & strategic AI integration. ^2^

Digitalization strategies allow small-scale industries to overcome environmental barriers. Digitalization strategies allow small-scale industries to overcome environmental barriers. Previous studies ^23^ demonstrate that mapping internal and external business factors is crucial for formulating effective digital strategies that address human resource limitations and market gaps in the MSME sector. These evolving dynamics across different crisis periods are systematically mapped in Table 2.

To provide a comprehensive overview of the research landscape, Table 2 synthesizes the thematic shifts in strategic innovation and SMI clusters across different timeframes, from the pre-pandemic era to the current recovery phase. Based on the synthesis presented in Table 2, while various aspects of SMI innovation have been explored, there is a clear need for a longitudinal mapping of global research trends. This gap justifies the use of bibliometric methods to provide a broader perspective on the evolution of this field.

## Results and discussion

### The development of research publications on strategic innovations in small and medium industries (SMIs) centres related to regional tourism from 2005 to 2025

The development of research publications on strategic innovations in Small and Medium Industries (SMIs) centres related to regional tourism from 2005 to 2025 The collected bibliometric data show a very positive and dynamic trend in research regarding strategic innovations in SMI centres that have contributed to the competitiveness of regional tourism over the past two decades. As summarized in Figure 2, from 2005 to 2025, there were 195 papers spread across 125 publication sources, demonstrating the diversity and wide dissemination of research in various reputable journals and conferences. The average annual growth of 15.86% indicates that this topic is getting significant attention from the academic community and practitioners. This figure indicates that strategic innovation in the centre of SMIs and its relation to regional tourism development is a rapidly growing research field relevant to the sustainable local economic development needs.

**Figure 2.**
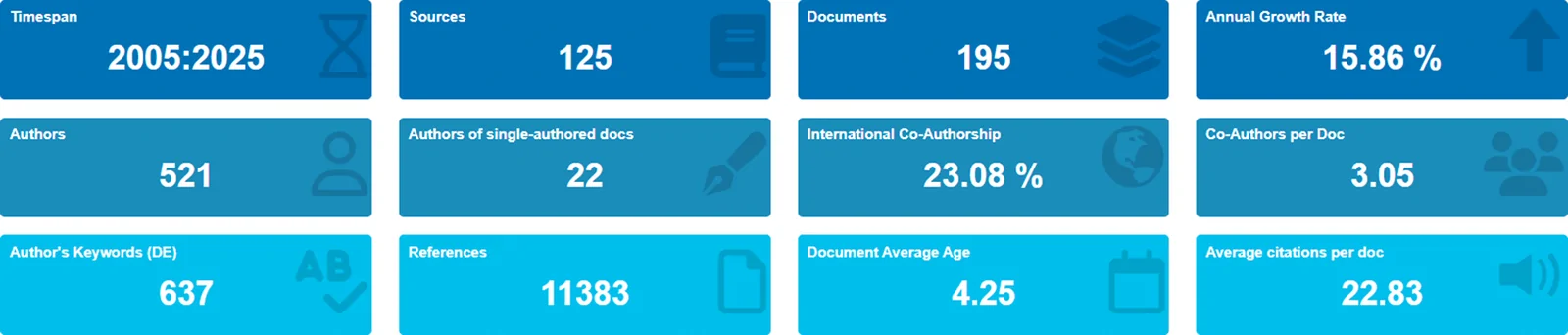
Development of Research Publications during the period 2005–2025 related to strategic innovations in small and medium industry centres and regional tourism.

The number of authors involved reached 521, with an average of 3.05 authors per document, reflecting intense collaboration between researchers. Nonetheless, only 22 papers were single works, suggesting that the research was largely collaborative, which typically resulted in a more comprehensive quality and scope of studies. In addition, the international collaboration rate of 23.08% indicates cross-border engagement that enriches the perspective and validity of research, while opening up opportunities for global knowledge transfer to local contexts. The authors use 637 unique keywords in terms of content, indicating a diversity of themes and broad research focusing on strategic innovation and regional tourism. The number of references reaching 11,383 shows that this research is built on a strong and scientifically interconnected literature foundation.

The average age of the documents is 4.25 years, indicating that most of the research is still relatively new and reflects recent developments in this area. This is also reflected in the high average citations per document, which is 22.83, indicating that these works have a significant influence and become an important reference in developing knowledge related to strategic innovation in the centre of SMIs and regional tourism. Overall, this data illustrates that research on strategic innovations in SMI centres that support regional tourism competitiveness is developing quantitatively and showing quality and collaboration that encourages the advancement of science and practice in this field. These positive trends serve as a solid basis for the development of further studies and the practical implementation of innovations in the context of local economic development and sustainable tourism.

### Leading authors, institutions, and countries have contributed significantly to strategic innovation research in regional SMI’s and tourism centres

Figure 3 shows a visualisation of the authorship network that illustrates the collaborative relationship between writers who are active in strategic innovation in the centre of SMIs and regional tourism. This network shows the concentration of a strong and interconnected group of authors, reflecting the intensive collaboration and intellectual synergy that drives research development in this field. Some of the author's prominent names, such as Zhang J., Ebrahimi A.H., Pulido-Fernández J.I., and Ramón-Rodríguez B., are seen as the centres of the main clusters with a high number of connections, signifying their central role in building research networks and producing influential works. These clusters indicate that research in strategic innovation and regional tourism is not only carried out individually, but is more based on cross-disciplinary and cross-institutional team collaboration. In addition, the network also reflects the geographical and institutional diversity of the contributors, suggesting that the research is multidimensional and involves a wide range of academic perspectives from different countries.

**Figure 3.**
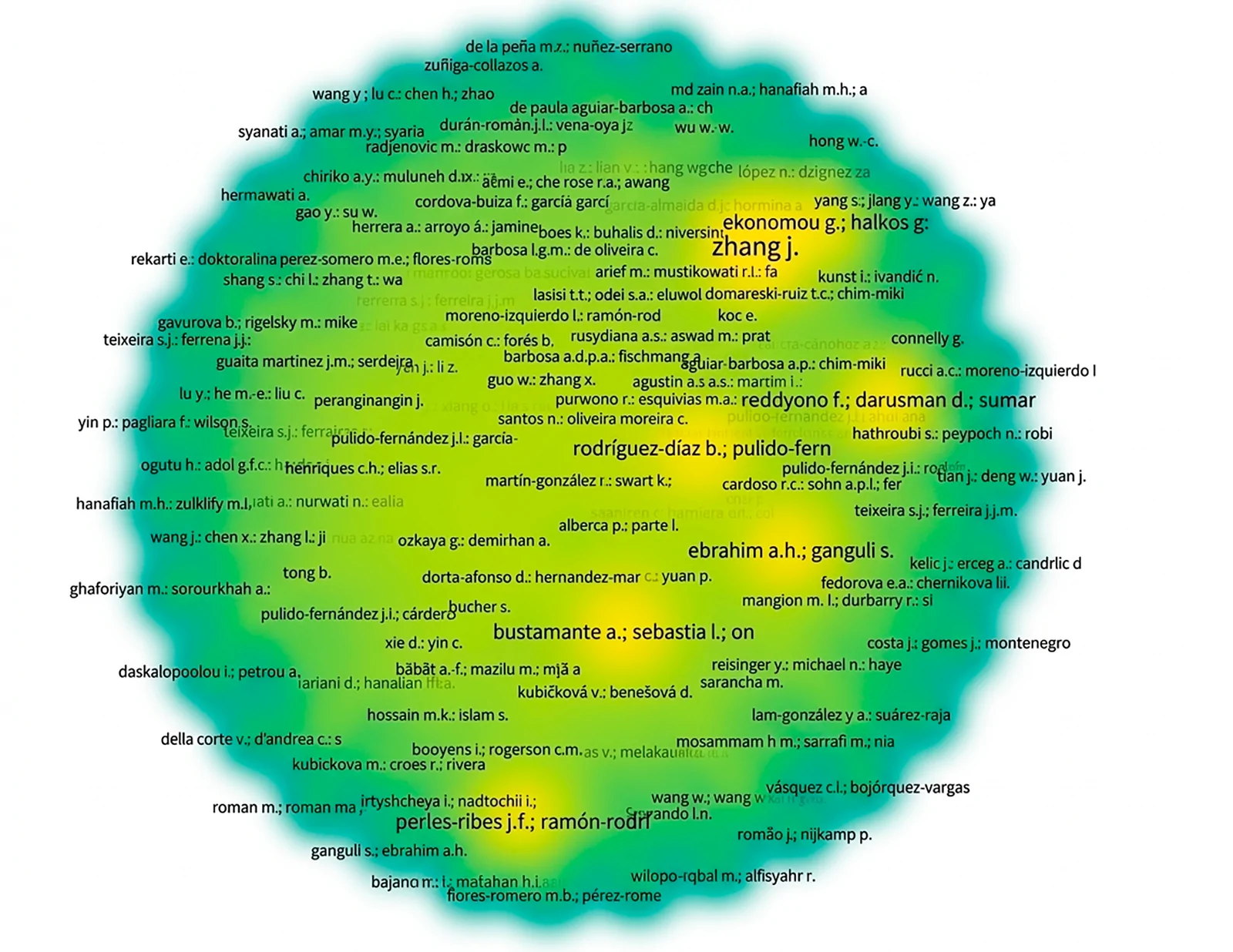
Authorship network.

This is important to enrich the quality and relevance of research results, especially in the context of globalisation and complex local challenges. The international collaboration established in this network strengthens the transfer of knowledge and technological innovations that can be applied effectively in SMI centres and regional tourism destinations. Thus, this strong authorship network not only accelerates the development of science but also supports the implementation of more adaptive and sustainable innovation strategies. Overall, this authorship network analysis confirms that leading researchers and key institutions have actively advanced strategic innovation in regional SMI and tourism centres. The established collaboration is an important foundation for the development of quality research and a positive indicator for the future of research and policy development based on scientific evidence.

Analysis of the Author’s Citation Network in Strategic Innovation Research at the Center for SMIs and Regional Tourism. The visualization in Figure 4 illustrates the structure of a network of quotes between authors that is very informative in identifying key figures and patterns of intellectual influence in strategic innovation related to SMI centers and regional tourism development. In the visualization, several authors such as Boes K., Buhalis D., and Inversini A. appear as the main center of gravity with a large node size, indicating the high number of citations they receive and a significant influence on the development of science in this field. The strong connection between these three authors shows that their works are important references and theoretical and empirical foundations in strategic innovation and tourism research. For example, Buhalis D. is widely known for his contributions to developing *the concept of smart tourism* and the digitalization of tourist destinations, which are very relevant to innovations in SMI centers that support the tourism sector. In addition, this network shows the existence of interconnected citation clusters, reflecting intellectual and constructive collaboration between authors. Authors such as Pulido-Fernández J.I. and Rodriguez also occupy strategic positions within the network, demonstrating their contributions in expanding the scope of research and enriching strategic innovation perspectives.

**Figure 4.**
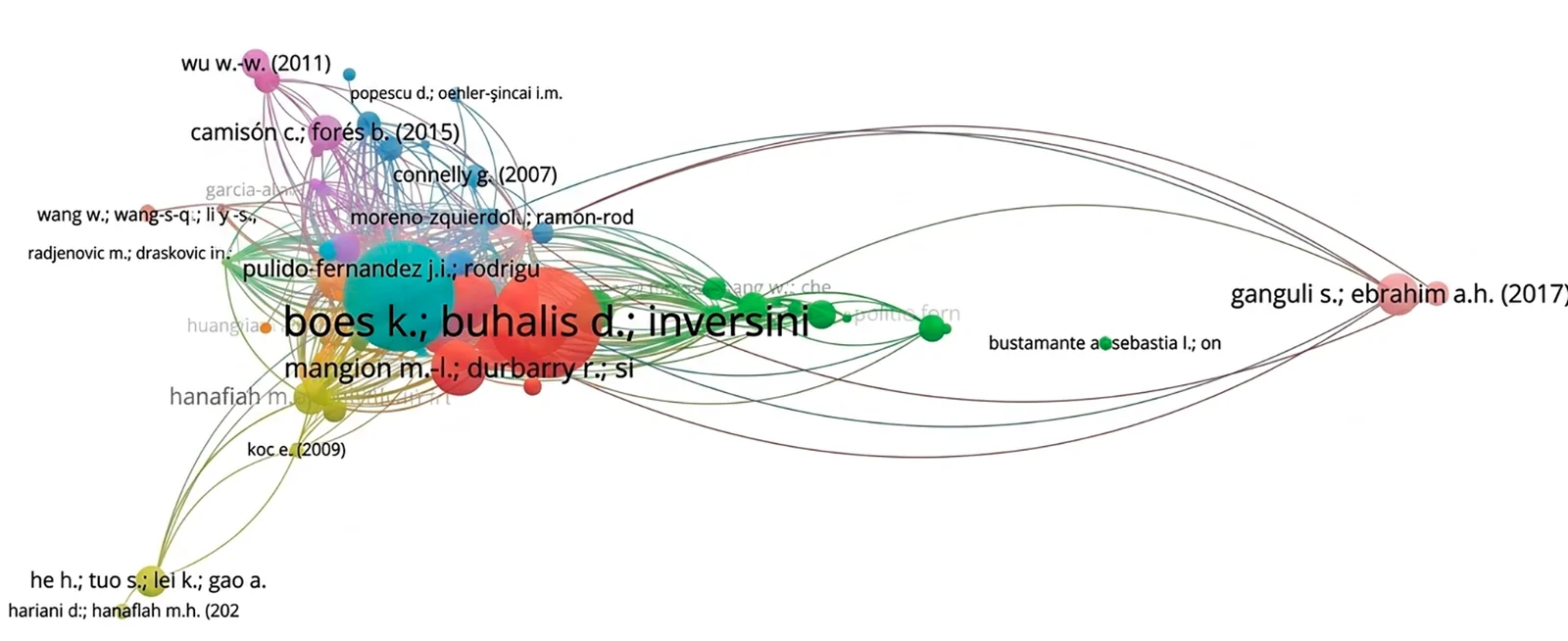
Co-authorship network.

Interestingly, there are relatively separate nodes such as, ^24^ which, although more isolated, still have a significant influence in certain contexts, indicating the existence of specific subtopics or research approaches but contributing to the development of science as a whole. This network of citations not only showcases key intellectual actors, but also illustrates the dynamics of knowledge dissemination and how strategic innovation ideas develop and transform through academic interactions. This visualization provides a clear picture of how the research community in this field is structured, and is an important tool for identifying collaboration opportunities as well as future research directions. Overall, this network analysis of citations confirms that strategic innovation research in regional SMI and tourism centers is supported by a strong literary foundation with contributions from a number of influential authors who have significantly shaped and directed the development of this field.

The bibliometric mapping results in Figure 4 align with the theoretical evolution previously synthesized in the literature matrix ( Table 2). The visualization shows a clear transition in research clusters, where early nodes focus on industrial efficiency, while more recent clusters are dominated by terms such as 'Strategic Innovation' and 'Sustainable Tourism.' This outcome validates the conceptual shift from a normal pre-pandemic focus to the innovation-driven recovery patterns identified in the literature review. Figure 5 illustrates the collaboration network between institutions active in strategic innovation research in Small and Medium Industry centres and regional tourism development. The visualisation shows the diversity and geographical spread of the institutions involved, ranging from universities, faculties, departments, to research centres across various countries and regions. The colours on the institutional nodes reflect the time of publication, with a spectrum from 2018 to a projection of 2024, indicating the dynamics and continuous developments in the institution's contribution to the field. Institutions in yellow indicate newer and more active publication activities, indicating an increased focus and investment in research on strategic innovation and SMI-based tourism in recent years.

**Figure 5.**
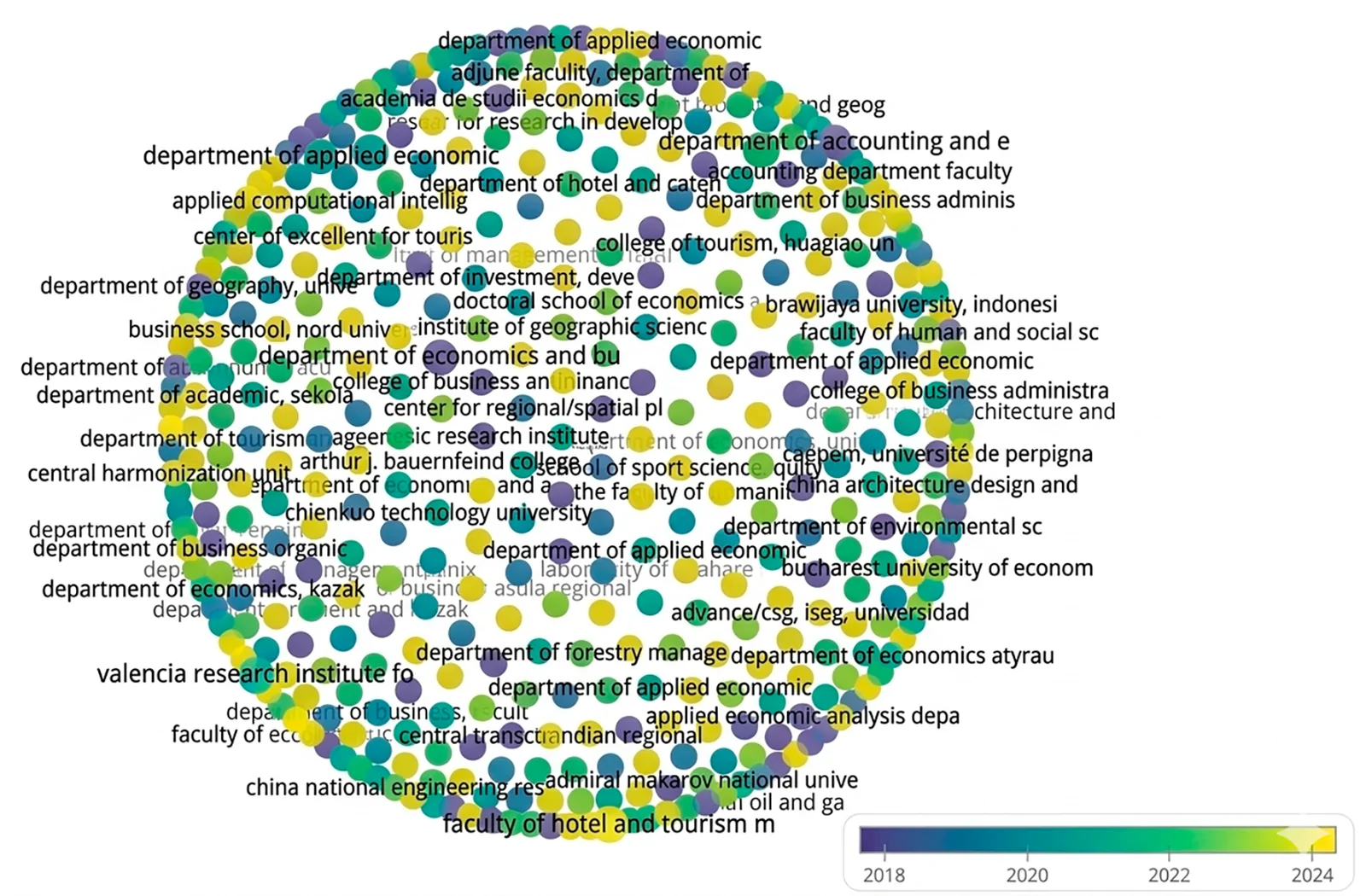
Organisations in the world that contribute significantly to strategic innovation research in regional SMI and tourism centres.

Several prominent institutions such as “Brawijaya University, Indonesia”, “China National Engineering Research”, “Chienkuo Technology University”, and “Valencia Research Institute” show a central role in this network. The presence of institutions from various countries confirms that strategic innovation research in regional SMI and tourism centres is a global issue with cross-border relevance. This network also indicates the existence of multidisciplinary collaborations involving various departments and faculties, such as economics, business, tourism, engineering, and social sciences. This multidisciplinary approach is essential to address the complexity of strategic innovation and sustainable tourism development challenges.

Overall, this visualisation confirms that the development of research in strategic innovation in the centre of SMIs and regional tourism is supported by a vast and dynamic network of institutions. This strengthens academic quality and breadth of perspectives and encourages international collaboration that can accelerate knowledge transfer and practical innovation in the field. This analysis provides a clear picture of how leading institutions play a role as centres for developing science and innovation while opening up opportunities for more intensive collaborative research. Thus, this study emphasises the importance of institutional support and global networks in strengthening the competitiveness of regional SMIs and tourism centres through strategic innovation.

The bibliometric clusters highlighting 'Competitiveness' and 'Service' underscore the importance of operational excellence in regional industry hubs. This is consistent with empirical findings by, ^25^ who demonstrated that product and service quality are the primary drivers of customer satisfaction in regional business clusters, such as those found in Bogor. Therefore, improving these variables is essential for SMIs to sustain their role within tourism-integrated clusters.

Figure 6. This shows the pattern of collaboration between institutions involved in strategic innovation research in the centre of SMIs and regional tourism development. This network visualisation illustrates the relatively focused interconnectedness between several key institutions, which shows the existence of research groups that interact intensively with each other. A cluster of closely interconnected institutions is on the left of the image, which most likely represents research centres or universities active in economics, tourism, and innovation. The strong linkages between institutions such as the tourism research institute, the international economics institute, the university institute of tourism, and the economics department show that multidisciplinary collaboration is a hallmark of this study. This is important because strategic innovation in the context of SMI and tourism centres requires an approach that integrates economic, management, and tourist destination development aspects.

**Figure 6.**
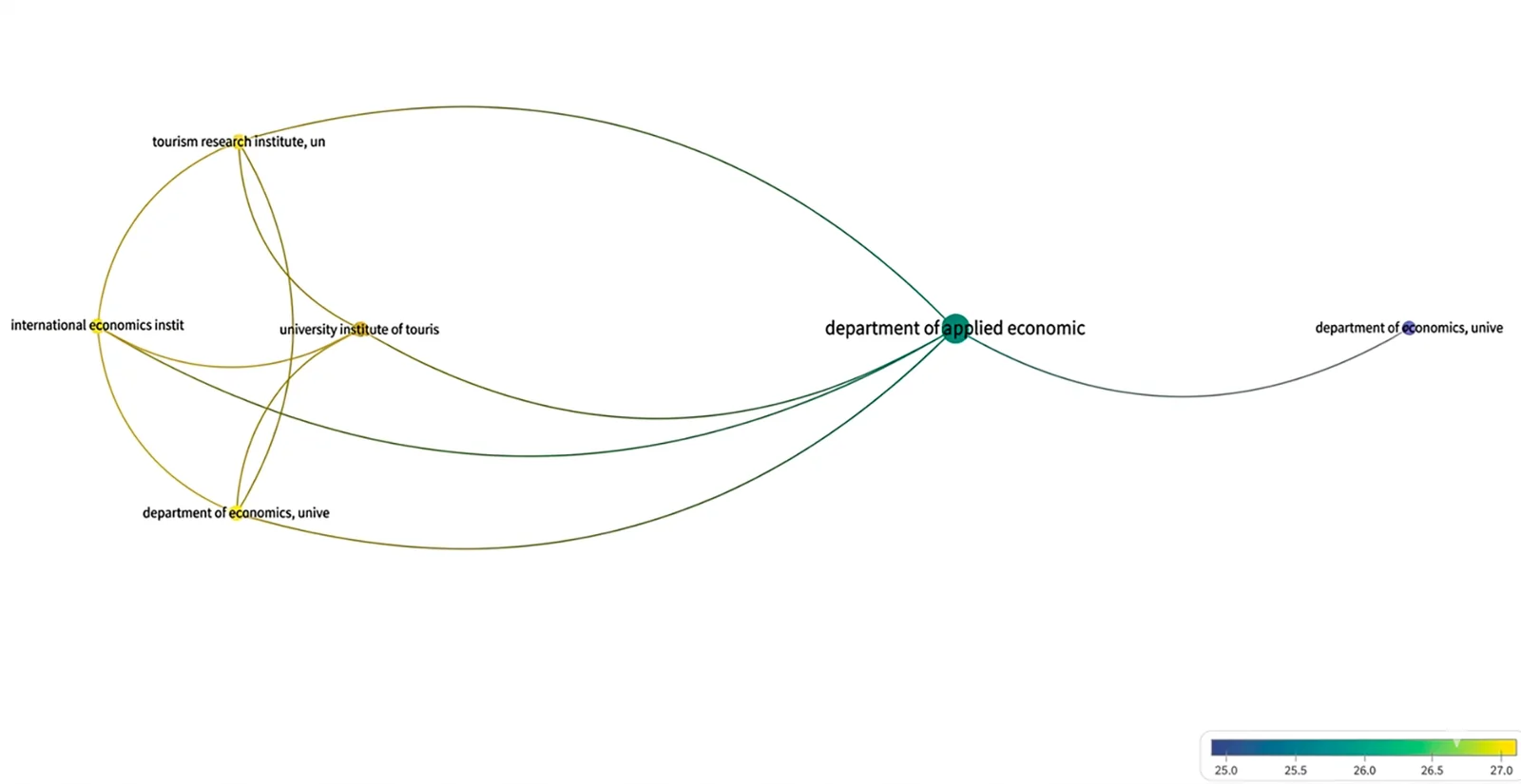
Organizational network.

The “Department of Applied Economics” occupies a central position in this network and links the institutional cluster on the left and the more isolated nodes on the right. This position signifies the strategic role of the institution in facilitating collaboration across research groups and expanding the reach of academic networks. This connectivity also signifies knowledge transfer and integration of perspectives that contribute to developing more comprehensive and applicable innovations. Although some institutions appear to be more isolated, the network reflects a relatively cohesive and structured pattern of collaboration, which is an essential foundation for developing quality and impactful research. The partnership between these institutions not only strengthens research capacity but also opens up opportunities for synergy in implementing strategic innovations that can sustainably increase the competitiveness of regional SMIs and tourism centres.

The bibliometric analysis reveals that strategic positioning and internal capability are key nodes in the current research trend. This aligns with the framework of, ^26^ who emphasize that a precise analysis of internal and external factors is vital for small enterprises to formulate development strategies that capitalize on location advantages and market opportunities. By identifying these factors, SMI clusters can better align their operational strengths with the demands of regional tourism. Thus, this visualisation confirms that a solid and multidisciplinary network of institutions supports the development of strategic innovations in regional SMIs and tourism centres. This is a key asset in facing complex and dynamic challenges in the sector, while encouraging the optimal use of academic resources for innovation and local economic development.

The analysis of the Inter-State Collaboration Network in Strategic Innovation Research at the Centre for SMIs and Regional Tourism, as summarised in Figure 7, shows the research collaboration network between countries active in strategic innovation in the centre of SMIs and regional tourism development. The visualisation depicts the strong and diverse relationship between countries, signifying that this topic has high global relevance and is of cross-continental concern. Countries like Spain, China, Indonesia, and Portugal are key hubs in this collaborative network, with large node sizes and many connections to other countries. This shows that these countries are not only actively producing publications but also have a central role in building international collaboration networks that strengthen the quality and impact of research.

**Figure 7.**
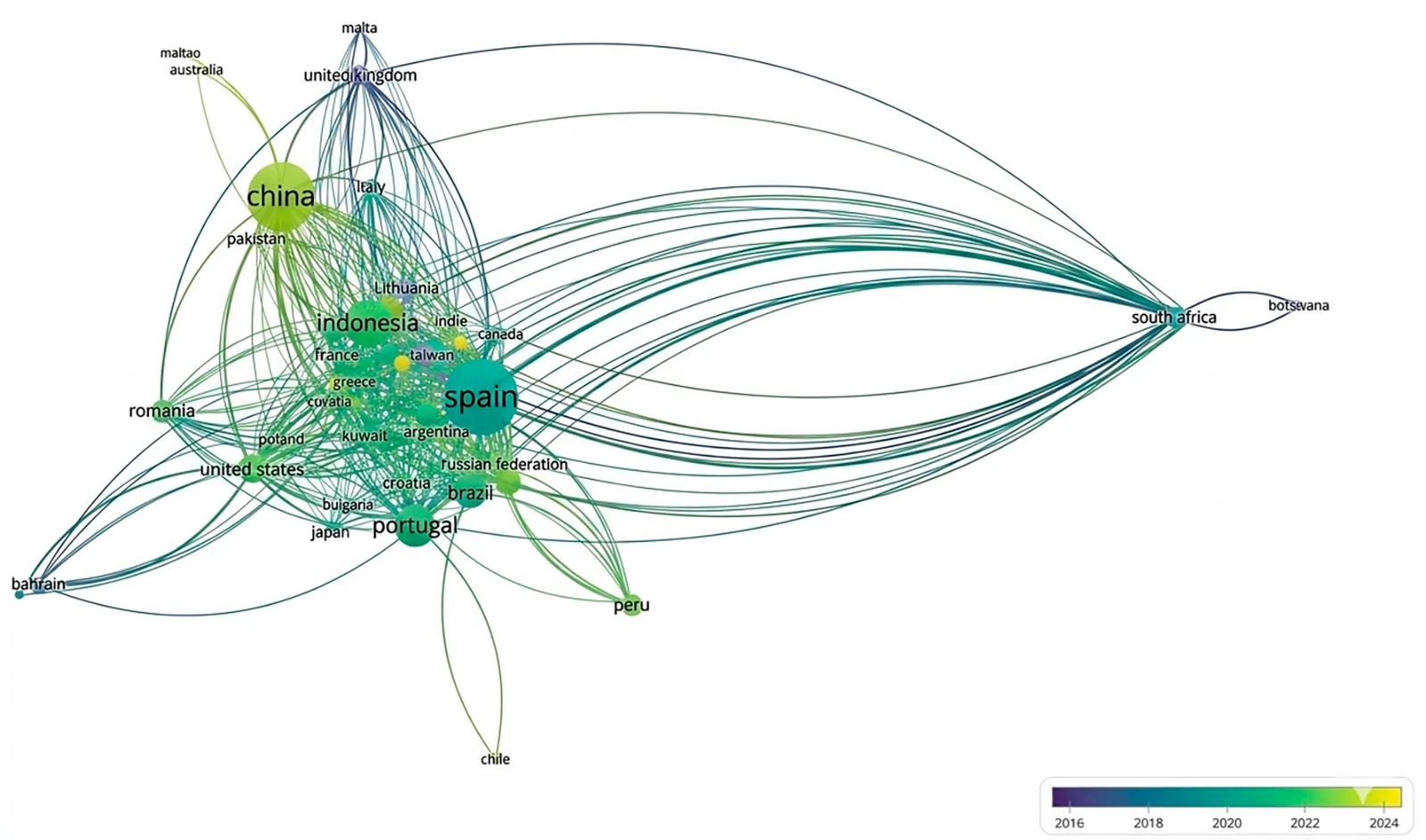
Countrywide network.

As the focus of this research, Indonesia appears to be an essential actor integrated into the global network, showing active involvement in international research collaborations. This emphasises that strategic innovation in the centre of SMIs and regional tourism development in Indonesia does not stand alone but receives support and knowledge exchange from the global academic community. In addition, the connection with countries such as Brazil, Peru, South Africa, and several other European countries shows a cross-continental and multidisciplinary collaboration network. This collaboration is essential to enrich research perspectives and expand the practical application of strategic innovation in diverse contexts.

The colours on the nodes that vary from 2016 to 2024 indicate the dynamics and development of collaboration that have continued and intensified in recent years. This trend suggests that research in this field is experiencing sustained growth and is adaptive to changing global needs and challenges. Overall, this network of collaboration between countries reflects a strong and mutually supportive research ecosystem, an essential foundation in developing strategic innovations in regional SMI and tourism centres. This international collaboration improves academic quality and accelerates the transfer of technologies and best practices that can be adopted to strengthen tourism competitiveness. sustainably.

### The dominant key and central theme in the literature are related to strategic innovation in the centre of SMIs to increase the competitiveness of regional tourism

As illustrated in Figure 8, the network visualization highlights the dominant keys and key themes that emerge in the literature related to strategic innovation, specifically showcasing how various concepts interact and form a comprehensive framework of thought in this field. The keyword network mapped in Figure 8 illustrates the main themes and linkages between key concepts in strategic innovation research that contribute to increasing regional tourism competitiveness. The keyword “competitiveness” emerged as the dominant centre with the largest size, indicating that competitiveness is the primary focus and central variable in this study. Around these key keywords, several essential clusters are closely interconnected, such as “innovation”, “tourist destination”, “sustainable development”, and “tourism industry”. This shows that innovation is not only seen as a technical process, but also as a strategic element that plays a role in the sustainable development of tourist destinations and the tourism industry.

**Figure 8.**
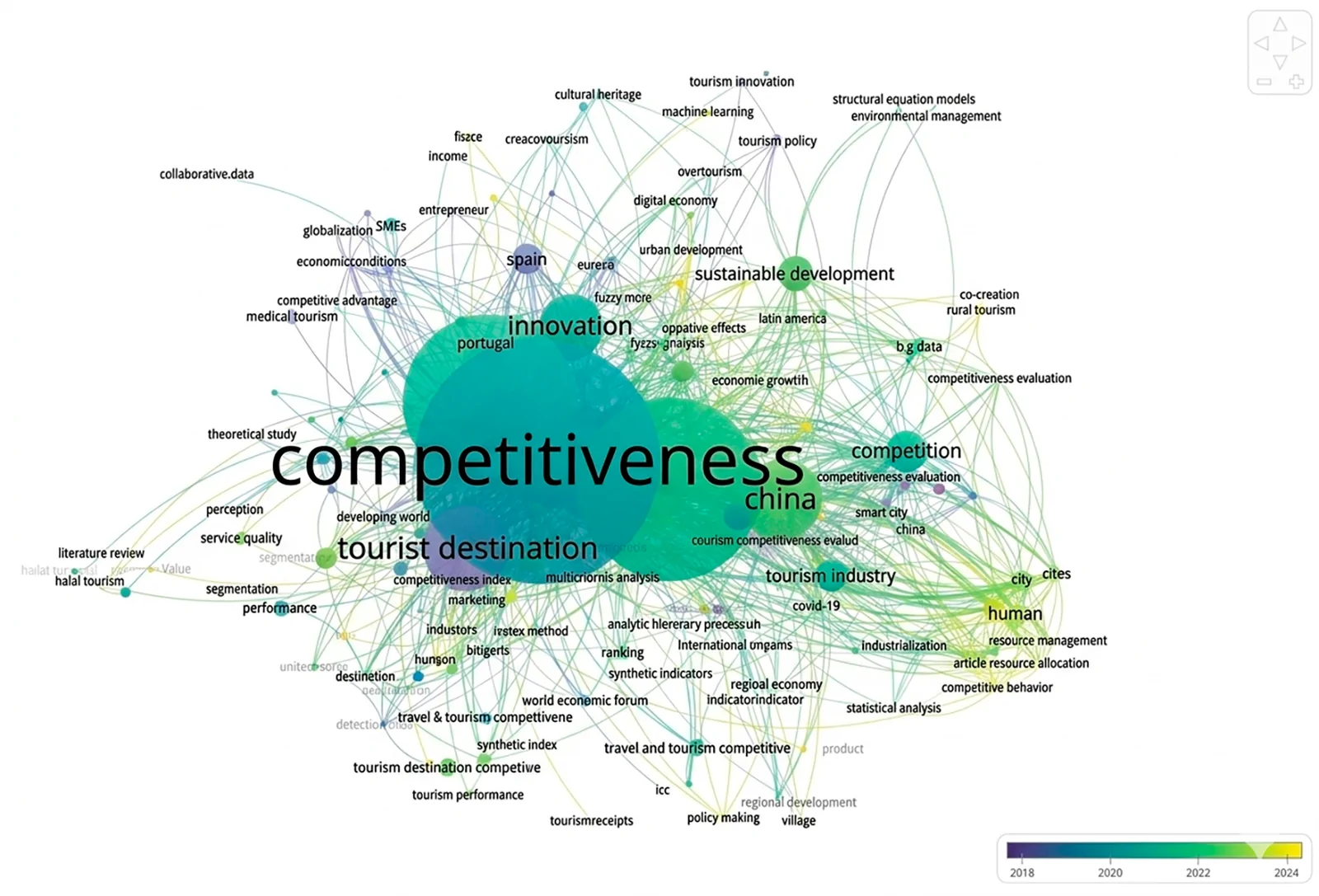
Dominant keys and key themes that emerge in the literature related to strategic innovation.

The prominent “sustainable development” cluster shows that sustainability is an essential concern in the latest research, which aligns with the global trend prioritising environmentally and socially friendly tourism development. This concept is also closely related to “environmental management” and “co-creation”, which signify a collaborative approach and responsible resource management as part of an innovation strategy. In addition, keywords such as “SMIs”, “digital economy”, “big data”, and “machine learning” reflect the integration of digital technology in the development of tourism innovation, which is increasingly strengthening as a driver of efficiency and a more personalised and interactive tourism experience. This is relevant to the need for SMI centres to adapt to digital transformation to increase the competitiveness of their products and services.

The “tourist destination” and “tourism industry” clusters emphasised that the research focuses not only on innovative products, but also on the complex aspects of destination management and the dynamics of the tourism industry. The association with terms such as “policy making”, “regional development”, and “competitive behaviour” indicates that market policies and behaviour are also an integral part of an effective innovation strategy. Overall, this network of keywords describes a multidimensional and interdisciplinary research landscape, combining economic, technological, environmental, and social aspects to improve regional tourism competitiveness through strategic innovation. This visualisation provides a rich picture of how various concepts interact with each other and form a comprehensive framework of thought in this field.

### A shared citation pattern and a bibliographic coupling network between documents, authors, institutions, and countries in the study

The comprehensive bibliographic mapping presented in Figure 9 highlights the interconnected network and evolutionary trends between documents, authors, institutions, and countries. Specifically, Figure 9 illustrates the cumulative development of key terms often used in the literature related to strategic innovation in SMI centres and regional tourism development over the past two decades. It can be seen that the term “competitiveness” occupies the most dominant position with very significant cumulative growth, especially since 2017. This confirms that competitiveness is the primary focus and central theme in research on strategic innovation in this sector. The terms “tourism” and “tourism competitiveness” also show a sharp upward trend, indicating that aspects of tourism and the competitiveness of tourist destinations are of great concern in the context of strategic innovation. The increased use of this term reflects the increasing awareness of the importance of tourism development as a key sector in the regional economy. In addition, the terms “innovation” and “sustainability” are experiencing stable and consistent growth, which shows that innovation is not only seen as a technical element but also an integral part of sustainable development. This is in line with global trends that demand the integration of environmental and social aspects in innovation strategies, especially in the tourism and SMI sectors.

**Figure 9.**
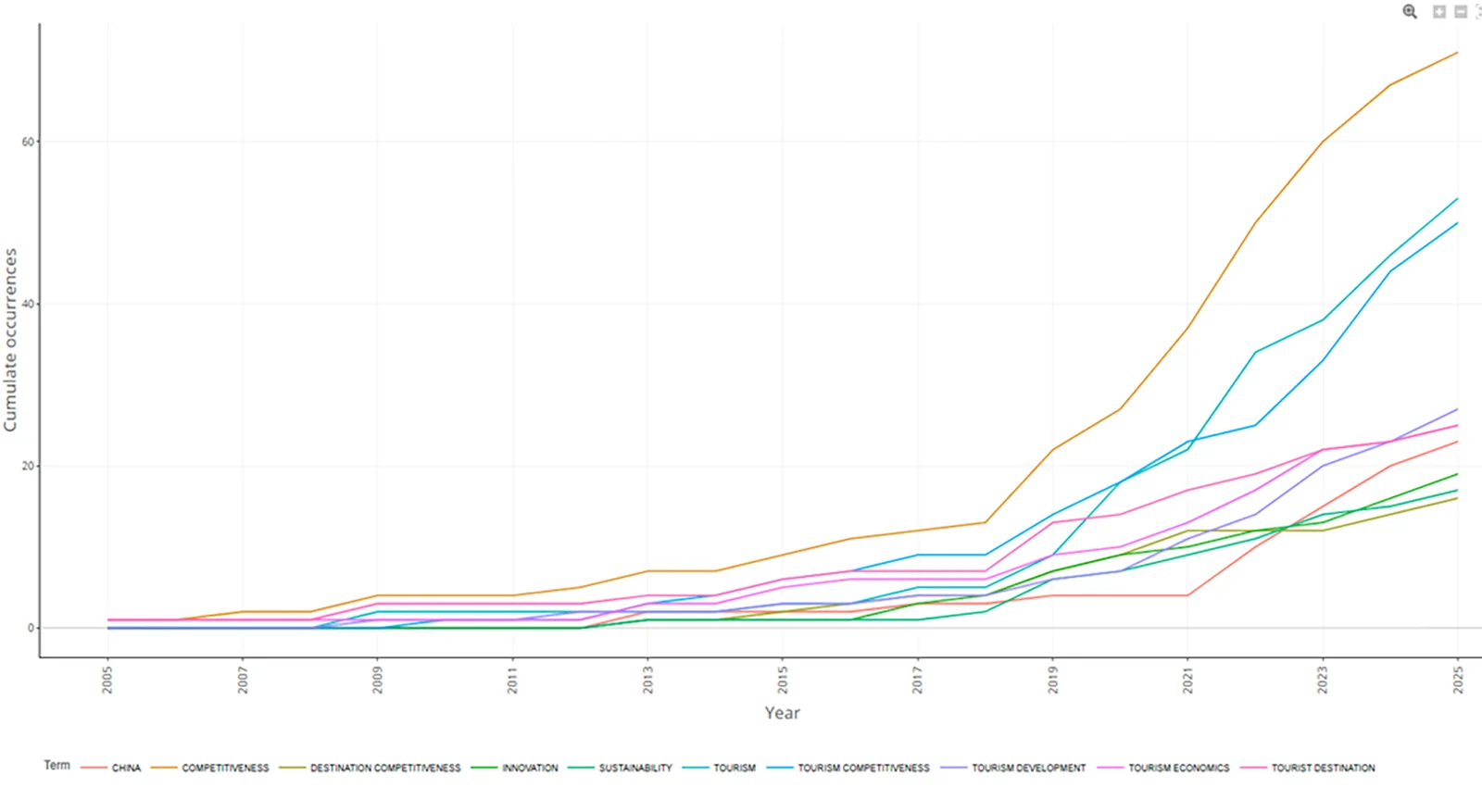
Bibliographic network between documents, authors, institutions, and countries.

Other terms such as “tourism development”, “tourism economics”, “tourist destination”, and “destination competitiveness” also showed a significant increase, indicating that studies related to the development of tourist destinations and the economic aspects of tourism are getting more attention in the academic literature. It reflects a multidimensional approach combining financial, social, and managerial aspects to develop strategic innovations. Interestingly, the term “China” also appears in the graph, albeit with more moderate growth, indicating that research involving contexts or case studies from China also contributes to the discourse of strategic innovation in regional SMIs and tourism centres. Overall, this graph confirms that research in strategic innovation to improve regional tourism competitiveness through SMI centres continues to grow with an increasingly sharp focus on competitiveness, innovation, and sustainability. This trend shows the maturity and diversification of research themes that will further enrich the development of theories and practices in the future.

Analysis of Collaboration of Publications Between Countries in Strategic Innovation Research at the Centre for SMIs and Regional Tourism. The data and structural mappings presented in Figure 10 reveal the pattern of collaboration of publications from various countries that contribute to strategic innovation research at the Centre for SMIs and regional tourism development. As demonstrated in Figure 10, China dominates the number of documents with the highest contributions, followed by Spain and Indonesia, which shows the central role of these three countries in developing research in this field.

**Figure 10.**
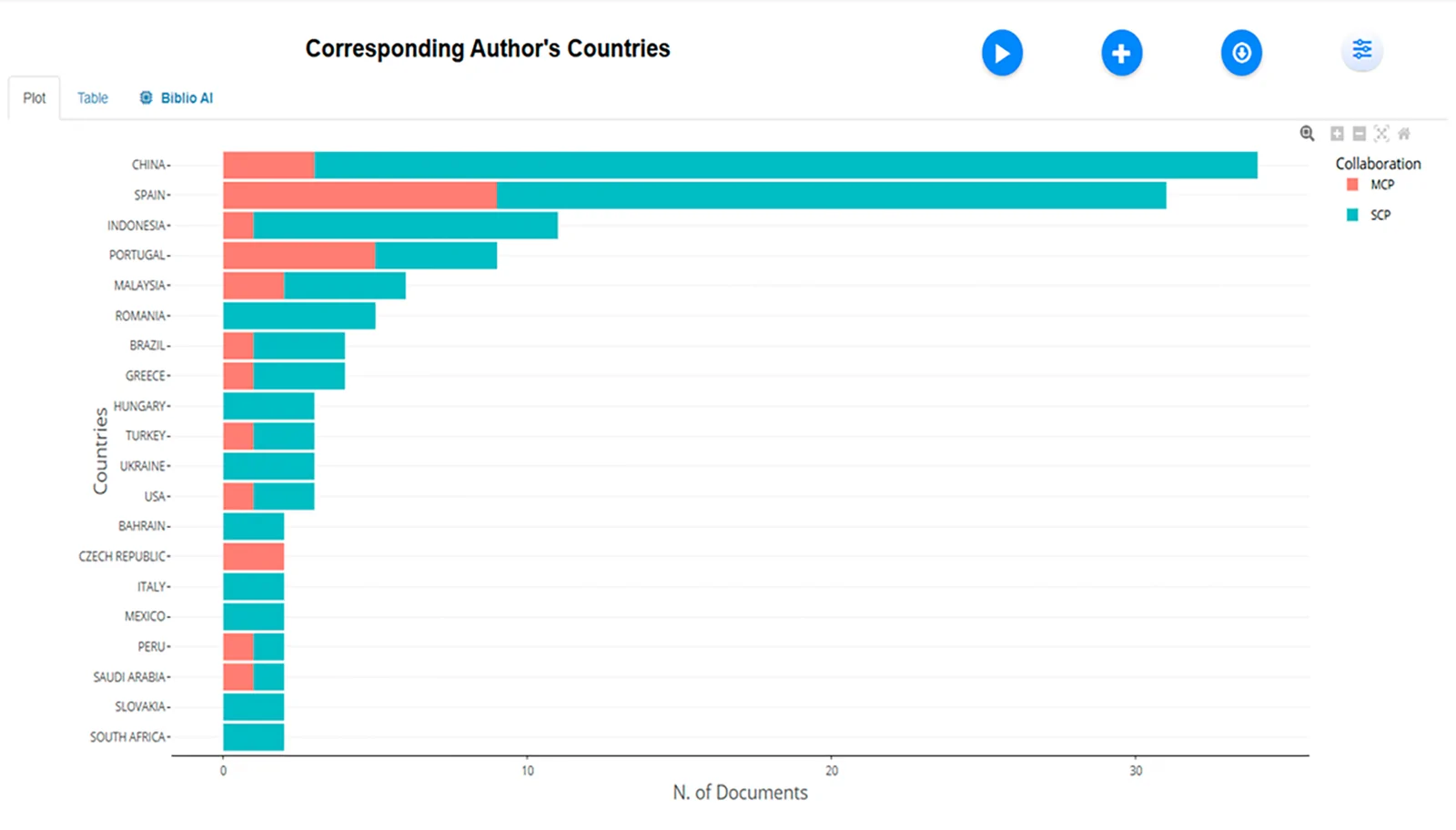
Bibliographic network between documents, authors, institutions, and countries.

China's significant contribution is seen in the relatively large number of single documents (MCP) and the dominating international collaborative documents (SCP). This indicates that research from China is strong nationally and active in establishing international collaborations that expand the influence and scope of research. Spain occupies the second position with many publications and a high proportion of international collaborations, reflecting its extensive research network and active involvement in the global academic community. As the primary focus of this research, Indonesia shows a significant contribution with a relatively high number of documents, especially in the form of international collaboration, which indicates openness and active participation in global research in strategic innovation and tourism.

Other countries such as Portugal, Malaysia, and Romania also show a growing role in the study, albeit with fewer documents. This distribution illustrates the geographical diversification in strategic innovation research, reflecting the global need and attention to developing SMI centres and tourism competitiveness in various regional contexts. The differences between MCPs and SCPs in some countries indicate the level of involvement in international collaboration. Countries with a high proportion of MCPs demonstrate the ability and willingness to collaborate across countries, which is a key factor in strengthening the quality and impact of research. In contrast, SCP-dominated countries may still focus on national research, which must be encouraged to expand collaborative networks. This data confirms that strategic innovation research in regional SMI and tourism centres is a dynamic field with broad global involvement. International collaboration is an essential driver in improving the quality of research and accelerating knowledge transfer, which will ultimately strengthen the development of innovation and tourism competitiveness sustainably.

Analysis of Network Citations, Authors, and Key Themes in Strategic Innovation Research of Regional SMI and Tourism Centres. The structural dynamics presented in Figure 11 highlight the evolution network of documents, authors, institutions, and keywords in strategic innovation development.

**Figure 11.**
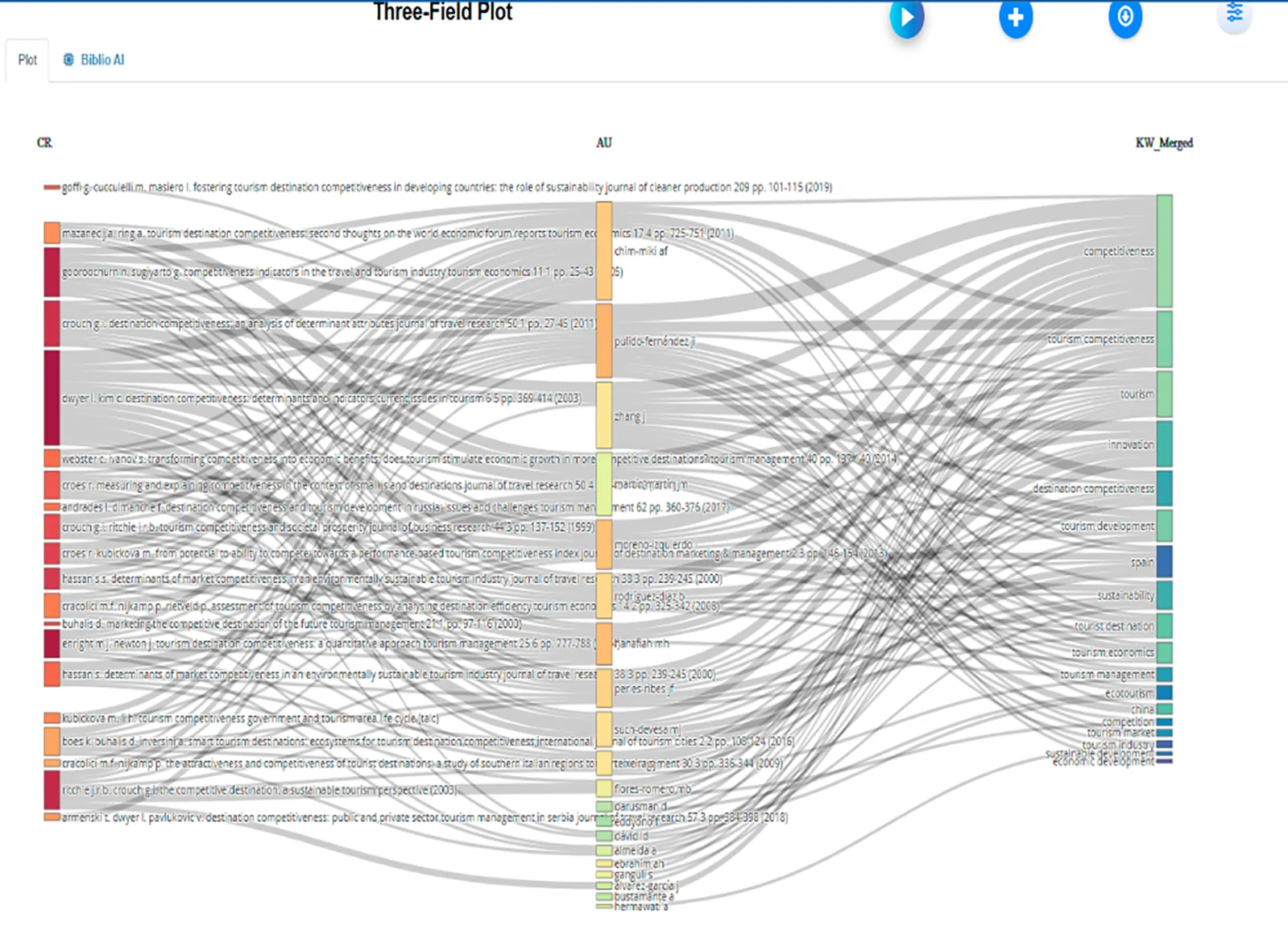
Evolution network of documents, authors, institutions, and keywords strategic innovation development.

On the left, the most cited documents have strong theoretical and empirical foundations, such as the work of ^27^ who discuss the role of sustainability in improving the competitiveness of tourist destinations in developing countries, and ^28^ who review the competitiveness of tourist destinations in the context of the World Economic Forum report. These documents are the central reference that directs and shapes the direction of research in this field. Authors such as Pulido-Fernández J.I., Zhang J., and Buhalis D. emerged as major contributors who linked various essential documents to the research theme. The presence of these authors as the centre of the network signifies their central role in developing strategic innovation concepts and models applicable in the context of tourism and SMIs.

The most dominant keywords included “competitiveness”, “tourism competitiveness”, and “tourism”, which indicated that the primary focus of the research was on improving the competitiveness of tourist destinations through innovation. In addition, keywords such as “innovation”, “destination competitiveness”, “sustainability”, and “tourism development” confirm that the aspects of innovation and sustainability are essential pillars in the development of effective strategies. The network also shows the close linkage between sustainability themes and innovation and competitiveness, reflecting research trends integrating environmental and social aspects into SMI-based tourism development strategies. This aligns with the global need for tourism, which is both competitive and ecologically and socially responsible. Overall, this analysis of the network of citations, authors, and keywords shows how the literature in the strategic innovation of regional SMI and tourism centres is built on a solid research foundation, with significant contributions from key authors directing research focus on key relevant and current themes. This visualisation emphasises the importance of integrating the concepts of competitiveness, innovation, and sustainability in shaping the future of sustainable and highly competitive regional tourism development research and practice.

The Analysis of the Development and Projection of Research Themes in the Field of Tourism Competitiveness, as detailed and summarised in Figure 12, describes the thematic evolution of research related to tourism competitiveness from 2005 to 2024 and the projected direction of research focus in 2025. The evolutionary pathway in Figure 12 highlights that key themes such as *tourism competitiveness* remain the primary focus and are expected to continue dominating the research landscape. This confirms that the development of the competitiveness of tourist destinations remains a crucial issue that is the primary focus of academics and practitioners.

**Figure 12.**
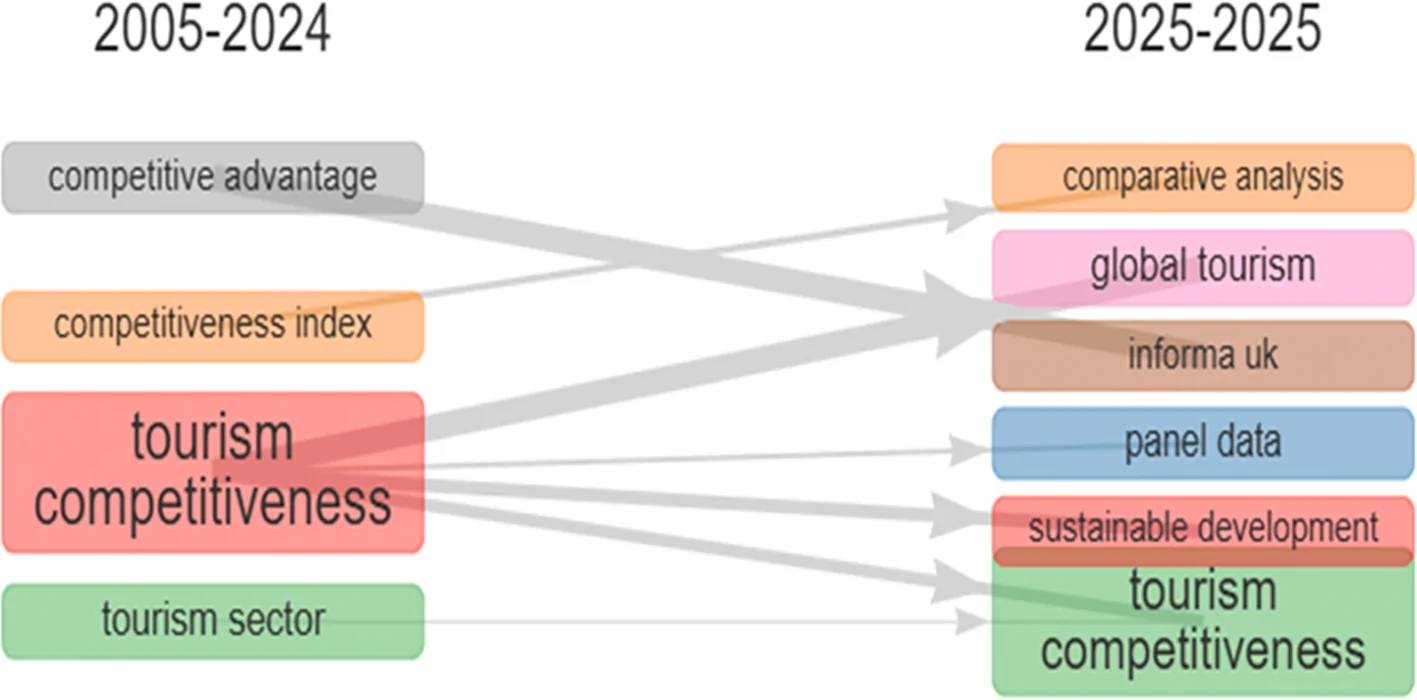
The evolution of strategic innovation in the centre of SMIs to increase regional tourism competitiveness.

In addition, the themes *of competitive advantage* and *competitiveness index* that dominated the previous period showed a shift in focus towards a more comparative and quantitative approach to analysis. By 2025, *comparative analysis* and *data panels* will emerge as increasingly popular analysis methods, signalling increased complexity and depth of studies that integrate longitudinal data and cross-contextual comparisons. The projection *of the global tourism* theme shows that the research will increasingly adopt a global perspective, considering that tourism is a cross-border phenomenon influenced by international dynamics. This requires a broader and holistic study to understand the competitiveness of tourist destinations in various parts of the world.

In addition, the theme is integrable sustainable *development. The research projection emphasises* a paradigm shift towards tourism development that is not only economically competitive, but also environmentally and socially sustainable. ^29^ ^,^ ^30^ This reflects the growing awareness of the importance of sustainability as an integral part of tourism competitiveness strategies. Overall, this data shows that research in the field of tourism competitiveness has undergone a significant transformation from focusing on simple indices and competitive advantages to a more comprehensive, quantitative, and globally insightful and sustainable approach to analysis. This trend reflects the maturity of the research field and readiness to face the complex, multidimensional challenges of the future.

### The contribution and implications of the results of this research to developing strategic innovations in s centres to increase regional tourism competitiveness

The results of the analysis show that there has been a significant increase in the number of publications related to strategic innovations in SMI centres that contribute to regional tourism competitiveness since 2010. *The dominant research themes include digitalisation, sustainability, innovative product development, and multi-stakeholder collaboration.* The most prominent countries and institutions in this research come from Southeast Asia, particularly Indonesia, with several international collaborations beginning to develop. Keyword analysis revealed that *“digital innovation”, “sustainable development”, and “tourism competitiveness*” were the primary focus of the research. The network of citations and bibliographic couplings shows the existence of interconnected research clusters, indicating a progressive consolidation of knowledge in strategic innovation in SMIs and tourism **.** This research's results significantly enrich academic understanding and strategic innovation development practices in Small and Medium Industries (SMI) centres, which drive regional tourism competitiveness. With a comprehensive bibliometric approach, this study successfully maps research trends, key actors, and key themes underlying the development of strategic innovation in the context of regional tourism over the past two decades.

The main contribution of this research lies in identifying the integration of digitalisation and sustainability aspects as the primary driver of strategic innovation that can increase the competitiveness of tourism based on SMI’s centres. These findings confirm that innovation must not only be technical or product-specific but must also include business model transformation, resource management, and cross-sector collaboration that is adaptive to market and environmental dynamics. ^31^ ^–^ ^36^ Practically, the results of this study provide a strong scientific basis for policymakers, SMIs, and regional tourism stakeholders in formulating and implementing more structured and effective innovation strategies. ^37^ ^–^ ^41^ The emphasis on multi-stakeholder collaboration and capacity building for digital innovation is a strategic recommendation that can accelerate the adoption of technology and sustainable practices at the local level. ^29^ ^,^ ^42^ ^–^ ^45^

In addition, this research opens up space for the development of evidence-based and contextual innovation models, which not only improve the economic performance of SMIs but also strengthen the regional tourism ecosystem as a whole Pérez-Romero et al., 2024). ^46^ ^,^ ^30^ This implication is significant in facing the challenges of globalisation, changes in tourist behaviour, and environmental and social sustainability demands. Thus, this study not only makes a theoretical contribution in enriching the literature on strategic innovation and tourism, but also provides a real practical impact in increasing the competitiveness and sustainability of regional tourism through strengthening SMI centres. ^47^ ^–^ ^51^ In the future, the results of this research can serve as a foundation for more in-depth empirical studies and the development of innovation policies that are responsive to local needs and global trends.

The findings of this study affirm the importance of strategic innovations that integrate digitalisation and sustainability as the primary drivers of increasing the competitiveness of tourism based on SMI centres. ^52^ ^–^ ^54^ The increase in publications and collaborations shows a growing awareness among academics and practitioners of the need for a holistic, innovative approach. However, cross-country and cross-sectoral collaboration must be strengthened to accelerate knowledge transfer and innovation capacity building. ^55^ The practical implications of this research emphasise the need for policies that support the digitalisation of SMIs, continuous innovation training, and the development of an inclusive and adaptive innovation ecosystem. This study also opens up opportunities for further research to explore the empirical implementation of innovation and evaluate its impact on local economic performance and tourism **.**

The findings of this study expressly affirm that strategic innovations that integrate digitalisation and sustainability aspects are the main drivers in increasing the competitiveness of tourism based on Small and Medium Enterprises (SMIs) centres. This is in line with the current literature that shows that digitalisation not only improves operational efficiency but also expands market access and enriches the personal experience of travellers. ^6^ ^,^ ^17^ In addition, the integration of sustainability principles in innovation is crucial to ensure that tourism development does not sacrifice natural resources and the social well-being of local communities. ^7^ ^,^ ^56^ ^,^ ^57^ ^,^ ^22^

The increasing number of publications and collaborations across institutions and countries identified in bibliometric analysis reflects the growing awareness among academics and practitioners of the need for a holistic and multidisciplinary innovative approach. Study by ^24^ ^,^ ^58^ ^–^ ^60^ emphasizing that collaboration and co-creation between various stakeholders is an important foundation in creating sustainable innovations that are relevant to market needs. However, the results of this study also show that cross-border and cross-sectoral collaboration is still relatively limited, which is an obstacle to accelerating knowledge transfer and innovation capacity building globally. ^4^ ^,^ ^5^ Therefore, strengthening international and cross-sector collaboration networks is a strategic priority to accelerate the diffusion of innovation and sustainably increase competitiveness. The synergy between strategic innovation and cluster development is a fundamental driver of regional prosperity. As demonstrated by, ^61^ entrepreneurial activities within specific agricultural and industrial sectors significantly contribute to broader economic growth in Indonesia, providing a baseline for how SMI clusters can stimulate local economies. This supports the bibliometric findings that emphasize the long-term impact of SMI development on regional stability.

From a policy perspective, the results of this study emphasise the importance of regulatory support and programs that encourage the digitalisation of SMI centres, including the provision of technology access, human resource training, and incentives for sustainable innovation. ^3^ ^,^ ^8^ ^,^ ^12^ ^,^ ^62^ ^–^ ^64^ The development of an inclusive and adaptive innovation ecosystem is also critical, where governments, the private sector, academia, and local communities play an active role in creating synergies that support sustainable and competitive innovation. ^10^ ^,^ ^11^ ^,^ ^12^ ^,^ ^65^ In addition, this research opens up opportunities for follow-up studies to explore the empirical implementation of innovation in the field and evaluate its impact on the performance of the local economy and the tourism sector. This kind of empirical research is needed to test the effectiveness of the proposed innovation strategy and to refine the innovation development model that is appropriate to the specific context of the SMI center and regional tourism, ^21^ ^,^ ^66^ ^–^ ^70^ these findings strengthen the understanding that strategic innovation that combines digitalization and sustainability is key to accelerating the transformation of SMI centers into the main drivers of regional tourism competitiveness. Multidimensional and collaborative approaches must be continuously developed to ensure innovation generates a competitive advantage and contributes positively to inclusive and sustainable economic development. ^71^ Establishing a recognizable identity is crucial for SMIs operating within tourism centers. In this regard, ^72^ highlight that brand experience and satisfaction are pivotal in building brand loyalty, which is mediated by trust. Integrating these branding elements into SMI strategic innovations can enhance the long-term competitiveness and sustainability of tourism-based clusters.

## Conclusion

This research has successfully mapped the evolution of strategic innovation trends within Small and Medium Industry (SMI) centers and their impact on regional tourism competitiveness over the past two decades. Through a systematic bibliometric approach, this study provides comprehensive insights into dominant research themes, influential actors, and evolving collaboration patterns. The findings confirm that digitalization and environmental sustainability are the central pillars of effective innovation strategies in the modern era. To maintain sustainable tourism competitiveness, it is recommended that stakeholders prioritize strengthening cross-sector collaboration and enhancing digital innovation capacity within SMI centers. These strategic steps are essential for bridging the gap between traditional practices and modern technological demands. Finally, further research should focus on empirical studies regarding the practical implementation of these innovations and the quantitative evaluation of their direct impact on the local economy.

The main findings confirm that digitalization and sustainability are the two fundamental pillars driving strategic innovation in SMI centers, while simultaneously strengthening regional tourism competitiveness within an increasingly fierce global context. This demonstrates that innovation transcends mere technological transformation; it represents the integration of sustainability values that foster inclusive and resilient local economic development. Strategically, this study emphasizes the necessity of strengthening multi-stakeholder collaboration—encompassing government, industry, academia, and local communities—alongside enhancing digital innovation capacity within SMI centers. Such measures are crucial to accelerating the adoption of adaptive innovations that remain relevant to evolving market demands and environmental challenges.

In the future, further research is strongly recommended to empirically examine the implementation of these strategic innovations and evaluate their impact directly on local economic growth and the sustainability of the tourism sector. This approach will enrich practical understanding while strengthening the scientific foundation for developing more effective innovation policies and strategies. Thus, this study makes a significant theoretical contribution and offers a practical guide for strengthening SMI centres as a driving force for sustainable and highly competitive regional tourism.

## Ethical considerations

As this study is a bibliometric analysis based on previously published academic literature, it did not involve human participants, clinical trials, or animal experimentation. Therefore, formal ethical approval was not required. The authors also declare that this research does not have any negative societal, ethical, or environmental impacts.

## Data Availability

The underlying data supporting this study is openly available on the Open Science Framework under the title “Strategic Innovation Trends in Small and Medium Industry Clusters for Enhancing Regional Tourism Competitiveness: A Bibliometric Analysis” at https://doi.org/10.17605/OSF.IO/8PMZK. ^73^ This repository contains the raw metadata extracted from the Scopus database (scopus_metadata.csv), which include all retrieved documents used for the bibliometric and systematic analysis. Data are available under the terms of the Creative Commons Zero v1.0 Universal (CC0 1.0) Public Domain Dedication OSF: Strategic Innovation Trends in Small and Medium Industry Clusters for Enhancing Regional Tourism Competitiveness: A Bibliometric Analysis. https://doi.org/10.17605/OSF.IO/8PMZK. ^73^ This repository contains the high-resolution VOSviewer network visualization maps and supplementary search string details. Data are available under the terms of the Creative Commons Zero v1.0 Universal (CC0 1.0) Public Domain Dedication OSF: PRISMA 2020 Checklist for 'Strategic Innovation Trends in Small and Medium Industry Clusters for Enhancing Regional Tourism Competitiveness: A Bibliometric Analysis'. https://doi.org/10.17605/OSF.IO/8PMZK. ^73^ Data are available under the terms of the Creative Commons Zero v1.0 Universal (CC0 1.0) Public Domain Dedication.
