# Strategic Innovation Trends in Small and Medium Industry Clusters for Enhancing Regional Tourism Competitiveness: A Bibliometric Analysis

**DOI:** 10.12688/f1000research.183398.2

**Published:** 2026-10-09

**Authors:** Christian Kuswibowo, Siti Jahroh, Alim Setiawan Slamet, Harianto Harianto

**Affiliations:** 1 IPB University, School of Business, Bogor, Indonesia; 2 Polytechnic APP Jakarta, Marketing Management Study Program, Jakarta, Indonesia; 3 IPB University, Faculty of Economics and Management, Bogor, Indonesia

**Keywords:** Strategic Innovation, Small and Medium Industries, Regional Tourism, Competitiveness, Sustainability, Bibliometric Analysis

## Abstract

**Objective:**

This bibliometric study evaluates the evolution of strategic innovation research within Small and Medium Industry (SMI) clusters to inform strategies for enhancing regional tourism competitiveness and long-term economic sustainability. Rather than merely synthesizing literature, this study maps the structural interplay between internal digitalization capabilities, collaborative networking, and cluster-based regional configurations, offering strategic insights for tourism-led regional growth.

**Methods:**

A systematic literature search was conducted using the Scopus database under the PRISMA framework. Articles were screened based on predefined inclusion and exclusion criteria, yielding a final dataset of 195 peer-reviewed articles published between 2005 and 2025. Bibliometric mapping and network co-occurrence analyses were executed using VOSviewer and the Bibliometrix R-package (Biblioshiny) to identify structural thematic clusters, author collaboration patterns, and temporal research trajectories.

**Results:**

The thematic mapping reveals a significant shift in research paradigms—transitioning from traditional cluster operations toward smart tourism integration, green innovation, and digital platform capabilities. Five primary thematic clusters emerged: (1) digital transformation in SMI clusters, (2) collaborative governance and regional network integration, (3) sustainable tourism practices, (4) destination competitiveness, and (5) innovation ecosystem configurations.

**Conclusion:**

The findings demonstrate strong thematic associations between digital adoption, sustainability frameworks, and regional tourism development. To effectively translate these research patterns into competitive advantages, regional policies must integrate cluster infrastructure with digital support frameworks and cross-sectoral governance.

## Introduction

Small and Medium Industries (SMIs) serve as a fundamental economic engine across regions in Indonesia, playing a pivotal role in employment generation, localized value addition, and regional economic resilience. ^1^ ^,^ ^2^ In the era of global economic integration and dynamic market shifts, the strategic positioning of SMI clusters has become increasingly intertwined with the expansion of the regional tourism sector. Strategic innovation—encompassing fundamental reconfigurations in products, processes, managerial practices, and business models—is crucial for enabling SMIs to adapt to volatile market demands and external disruptions. ^3^ Rather than operating in isolation, SMI clusters provide the supply chain foundation, cultural authenticity, and localized product ecosystems that directly enhance destination attractiveness and regional tourism competitiveness.

The rapid evolution of digital technologies and sustainable development imperatives has further transformed industrial and tourism landscapes. Digital transformation acts as a primary catalyst for SMI capability building, enabling small-scale enterprises to optimize internal operations, enhance market access, and engage in cross-sectoral collaboration. ^4^ ^,^ ^5^ Within tourism destinations, the integration of digital tools fosters ‘smart tourism’ ecosystems, enhancing visitor experiences and destination management efficiency. ^5^ ^,^ ^6^ Concurrently, global shifts toward circular economy practices and green innovation demand that SMI strategic innovations align economic objectives with environmental preservation and social welfare.

Integrations of product and process innovations in the tourism sector are vital for supporting long-term sustainable growth. ^7^ A collaborative approach through co-innovation underscores that synergy among business actors, government entities, and local communities is essential to create shared value and accelerate adoption processes. ^8^ Frameworks for national competitiveness provide a strong theoretical foundation for understanding how strategic innovation creates sustainable competitive advantages for SMIs and regional tourism destinations. ^9^ ^–^ ^14^ Furthermore, global trends prioritizing sustainability and circular economy principles align economic goals with environmental preservation, ^14^ ^–^ ^16^ while social media and digital technologies significantly enhance customer engagement and community-based tourism marketing. ^17^

However, innovation management faces structural challenges, particularly in overcoming organizational rigidity that hinders product adaptation. ^18^ To address these dynamics, this study systematically examines the development of strategic innovation trends within SMI clusters supporting regional tourism. A bibliometric approach was selected as an appropriate method to map publication patterns, key actors, dominant research themes, and collaborative networks over the past two decades (2005–2025). Ultimately, this structural mapping provides comprehensive insights and strategic recommendations to navigate digital transformation and sustainability demands in regional tourism development.

Although numerous studies have addressed industrial innovation and tourism development separately, a systematic synthesis capturing their structural interface remains limited. Existing reviews often focus narrowly on firm-level capabilities without systematically mapping the macro-level intellectual structure and thematic shifts that have evolved over time. To bridge this gap, this study conducts a comprehensive bibliometric analysis of publications from the past two decades (2005–2025) to uncover collaboration patterns, thematic transitions, and structural configurations in SMI clusters linked to regional tourism competitiveness. To guide the investigation systematically, specific research questions were formulated anchored in the Population, Intervention, Comparison, Outcome, and Context (PICOC) framework.

## Materials and methods

This study employs a systematic bibliometric analysis to map the structural development, thematic trends, and collaborative networks within the domain of SMI strategic innovation and regional tourism. Data collection was executed using the Scopus database, covering publications over a twenty-year period (2005–2025). The search query utilized Boolean logic combining terms related to strategic innovation, small and medium enterprises, competitiveness, and tourism destination dynamics: *((“strategic innovation” OR “innovation strategy”) AND (“small and medium enterprises” OR “SMIs” OR “small and medium industries” OR “SMI centers”) AND (“competitiveness” OR “competitive advantage” OR “market competitiveness”) AND (“tourism” OR “regional tourism” OR “local tourism” OR “tourism industry”)).* An initial search yielded 3,295 records. Refinement was performed based on predefined inclusion criteria: finalized peer-reviewed journal articles, written in English, and published under open access or hybrid gold models. Applying these parameters resulted in a final dataset of 195 relevant articles for visual network mapping and qualitative thematic synthesis.

Bibliometric visualization and quantitative mapping were performed using VOSviewer and the Bibliometrix R-package (Biblioshiny). These tools enabled the analysis of publication growth trajectories, author co-authorship networks, institutional affiliations, and keyword co-occurrence patterns. To ensure methodological rigor and reproducibility, the study design followed the Preferred Reporting Items for Systematic Reviews and Meta-Analyses (PRISMA) framework and incorporated the Population, Intervention, Comparison, Outcome, and Context (PICOC) structure (illustrated in Figure 1).

**Figure 1.**
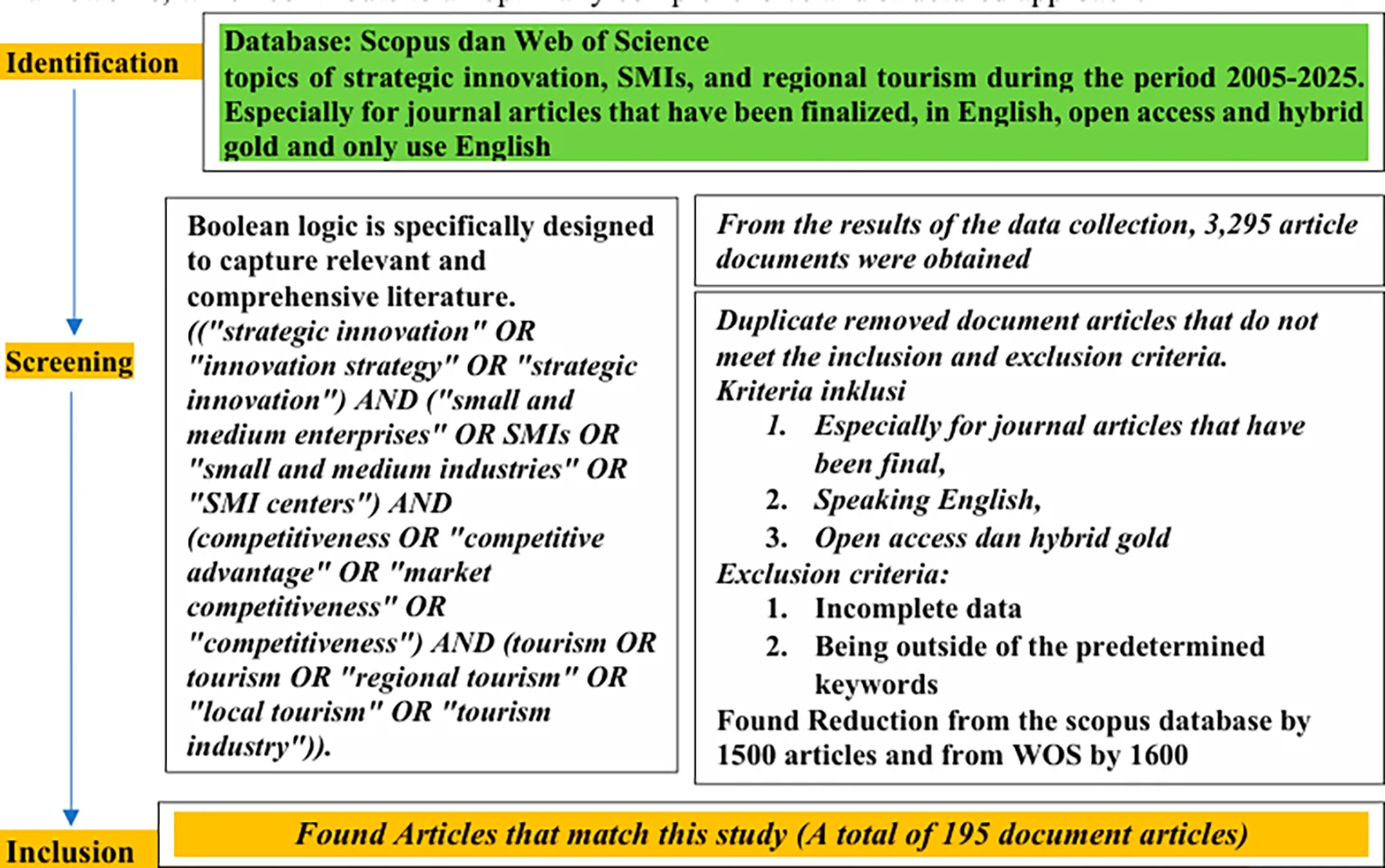
PRISMA view of the data filtering process.

The data selection procedure followed the Preferred Reporting Items for Systematic Reviews and Meta-Analyses (PRISMA) framework across three sequential stages: *Identification*, *Screening*, and *Inclusion* ( Figure 1). In the *Identification* stage, relevant literature published between 2005 and 2025 was retrieved from the Scopus database using the predefined Boolean search query, yielding an initial total of 3,295 records.

During the *Screening* stage, records were filtered based on structural alignment with the research scope, document type, and language criteria. Non-peer-reviewed items, duplicates, and articles published outside the open access or hybrid gold framework were excluded. Following this screening, a final set of 195 high-quality journal articles met all eligibility criteria and was retained for bibliometric mapping and qualitative thematic synthesis.

Following the data filtering process, 195 peer-reviewed articles met all predefined quality and eligibility standards. This final dataset served as the core corpus for visual network analysis and qualitative synthesis to uncover strategic innovation dynamics, key research clusters, and structural collaboration networks within SMI centers and regional tourism ecosystems.

To complement the bibliometric mapping and ensure rigorous analytical focus, the Population, Intervention, Comparison, Outcome, and Context (PICOC) framework was applied to structure the core research questions. The detailed operationalization of each PICOC component is summarized in Table 1.

**Table 1.** PICOC framework criteria and formulated research questions.

Initials	Component	Details of components	Formulation of research questions	Number RQ
P	Population	Small and Medium Industry (SMI) centers that contribute to regional tourism sectors.	How has the volume of scientific publications regarding strategic innovation in SMI centers evolved from 2005 to 2025?	RQ1
I	Intervention	Implementation of strategic innovation and digital transformation within SMI centers.	How does the implementation of strategic innovation contribute to enhancing regional tourism competitiveness?	RQ2
C	Comparison	Analysis of contributions and collaborative networks across different authors, institutions, and countries.	Who are the leading authors, institutions, and countries significantly contributing to this research field?	RQ3
O	Outcome	Identification of dominant research themes, citation patterns, keyword trends, and practical implications.	What are the central research themes and dominant keywords that define the landscape of strategic innovation in SMIs?	RQ4
C	Contex	Global and regional tourism dynamics from 2005 to 2025.	What are the citation patterns, co-citation networks, and bibliographic coupling structures within this context?	RQ5

Table 1 details the operationalization of the PICOC framework along with the corresponding research questions (RQ1 to RQ5). This structured approach ensures that the study remains focused and systematically addresses all critical dimensions of strategic innovation within the SMI and regional tourism sectors.

For data analysis, VOSviewer and RStudio (Biblioshiny) were utilized to perform bibliometric mapping and network visualizations across key units of analysis, including authors, institutions, and countries. The analytical scope encompassed co-authorship, co-occurrence, citation, co-citation, and bibliographic coupling structures, using the full counting method to provide a comprehensive mapping of the research landscape.

In interpreting the generated bibliometric networks, visual parameters were systematically evaluated. The physical distance between nodes reflects the strength of thematic or co-authorship relationships, while the node size corresponds to the publication volume or citation frequency. Furthermore, color variations denote distinct cluster groupings within the dataset.

By integrating these visual parameters with the structured PICOC framework, the analysis provides a rigorous, data-driven synthesis to address each formulated research question.

Technological innovation acts as a critical catalyst for sustainable economic growth. ^19^ As highlighted in recent systematic literature, integrating technological frameworks into industrial clusters and start-ups is essential for enhancing operational resilience and achieving broader economic development goals. ^20^

To establish a solid theoretical foundation prior to presenting the bibliometric visualizations, a systematic synthesis of seminal literature was conducted. This overview outlines core variables and key empirical findings that shape current understandings of strategic innovation within SMI centers. A summary of these foundational contributions is presented in Table 2.

**Table 2.** Matrix of research topics, innovation strategies, and regional focus across periods.

Variable/topic	Pre-pandemic (Normal)	During pandemic (Crisis)	Post-pandemic (Recovery/Future)
Regional Tourism	Focus on destination branding and infrastructure. ^21^	Focus on local safety and health protocols; travel collapse.	Sustainable, resilient, and smart tourism. ^22^
SMI Clusters	Industrial efficiency and labor-based clusters. ^1^	Supply chain vulnerability; shift to virtual collaboration.	Innovation-driven clusters and knowledge dynamics. ^13^
Strategic Innovation	Traditional product and managerial innovation. ^3^	Survival-based digitalization and business model shifts.	Green innovation and strategic Artificial Intelligence (AI) integration. ^2^

As summarized in Table 2, the evolution of research themes demonstrates a clear shift from traditional operational efficiency toward digital transformation, resilience, and sustainable innovation. Digitalization strategies enable small-scale industries to navigate environmental disruptions effectively. ^23^ Furthermore, mapping internal and external business capabilities remains essential for formulating targeted digital strategies that address human resource constraints and market gaps across the MSME sector.

While previous studies have examined isolated dimensions of SMI innovation, a comprehensive longitudinal mapping of global research dynamics remains limited. To bridge this knowledge gap, the following section presents a systematic bibliometric analysis based on the finalized dataset retrieved from Scopus, offering a structural perspective on publication trajectories, core thematic clusters, and international collaboration networks.

## Results and discussion

### Development of publication trends on strategic innovations in SMI centers and regional tourism (2005–2025)

The bibliometric data reveals a strong growth trajectory in scientific publications examining strategic innovations within Small and Medium Industry (SMI) centers and regional tourism over the past two decades. As illustrated in Figure 2, a total of 195 peer-reviewed papers were published across 125 distinct sources between 2005 and 2025, highlighting the diverse academic avenues addressing this topic. With an average annual growth rate of 15.86%, this domain reflects expanding interest among academic researchers and practitioners aiming to strengthen local economic resilience and sustainable regional development.

**Figure 2.**
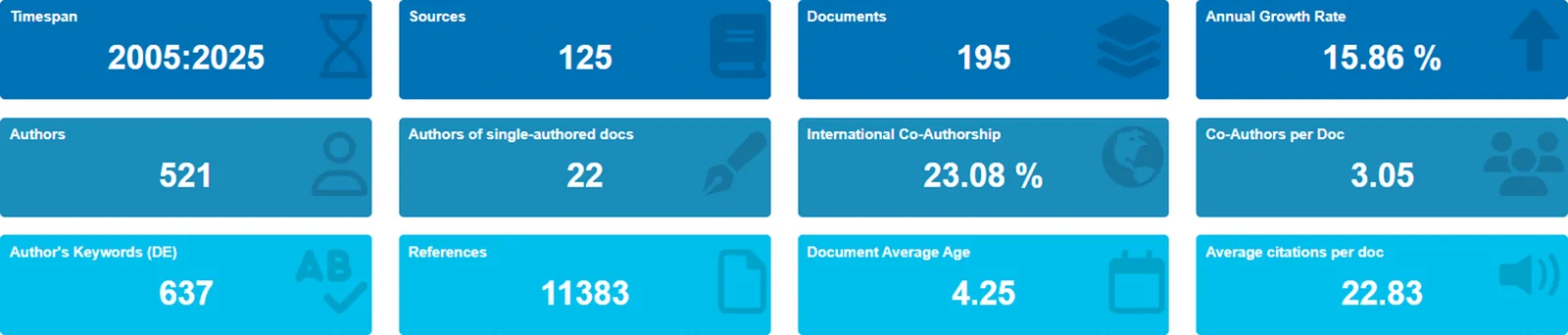
Main bibliometric indicators and overview of the publication dataset (2005–2025).

A total of 521 authors contributed to this field, with an average of 3.05 authors per document, demonstrating a strong collaborative dynamic among researchers. Notably, only 22 papers were single-authored, confirming that strategic innovation in SMI centers is predominantly examined through multi-disciplinary and multi-author collaborations. Furthermore, an international co-authorship rate of 23.08% points to meaningful cross-border engagement, facilitating global knowledge transfer to local industrial contexts. The identification of 637 unique author keywords highlights a rich diversity of sub-themes, while the reliance on 11,383 cited references underscores a robust, highly interconnected theoretical foundation.

Additionally, the average document age of 4.25 years indicates a contemporary literature body, reflecting rapid ongoing advancements in this domain. Coupled with a substantial average citation impact of 22.83 citations per document, these findings confirm that research in strategic innovation within SMI centers and regional tourism is not only expanding quantitatively but also yielding highly influential, scholarly contributions. Collectively, these positive indicators establish a strong foundation for future research and practical implementation in regional economic development.

### Leading authors, institutions, and countries contributing to strategic innovation in SMI centers and regional tourism

Figure 3 illustrates the co-authorship network representing collaborative relationships among key researchers in strategic innovation within SMI centers and regional tourism. The network displays distinct collaborative clusters, reflecting intellectual synergies that drive research in this field. Prominent authors—such as Zhang J., Ebrahimi A.H., Pulido-Fernández J.I., and Ramón-Rodríguez B.—emerge as central nodes with high link strength, signifying their pivotal roles in building research networks and producing impactful publications. These clusters confirm that research in this domain relies heavily on cross-disciplinary and cross-institutional teamwork. Furthermore, the geographical diversity of key contributors demonstrates the global scope and multidimensional nature of strategic innovation research.

**Figure 3.**
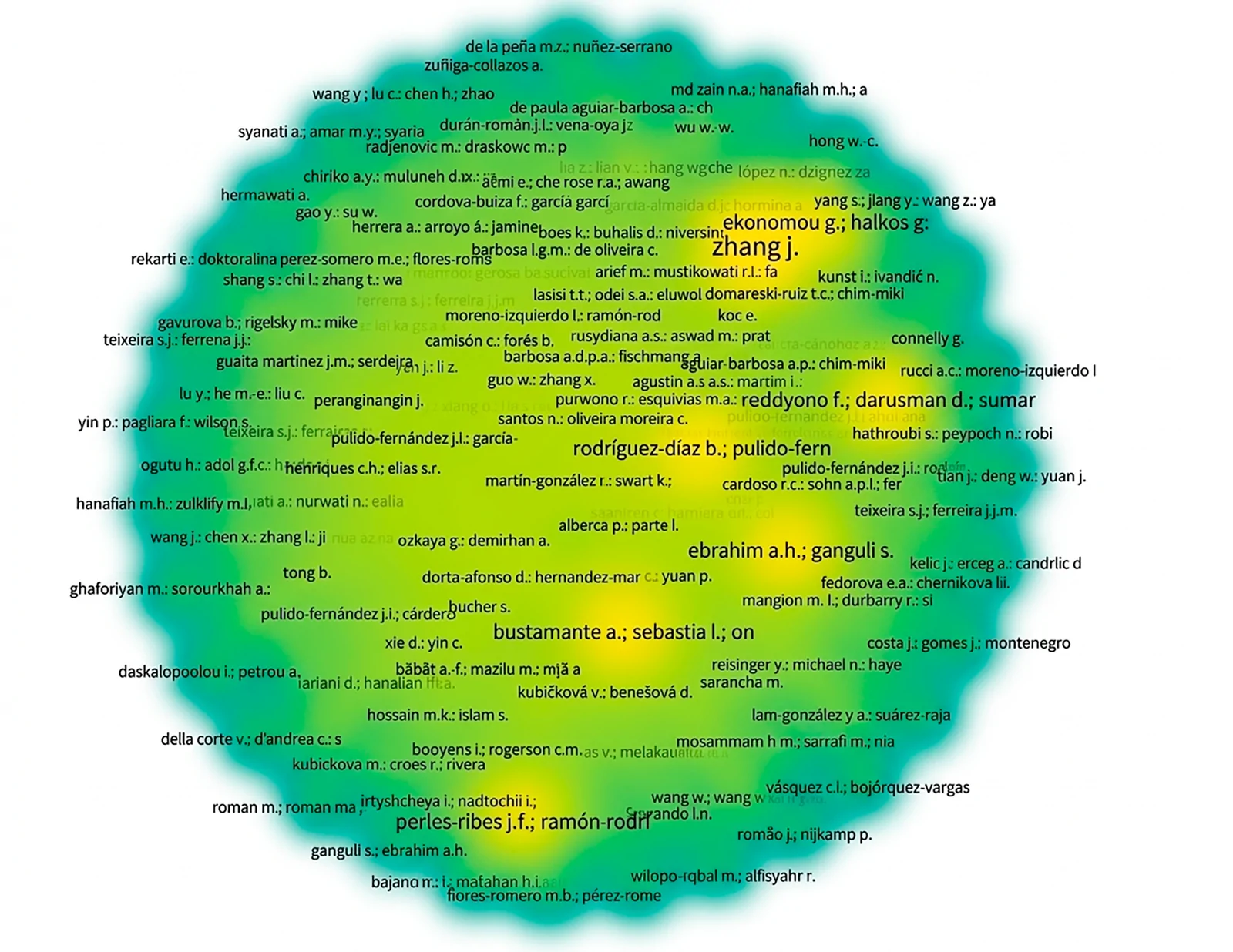
Co-authorship network visualization of active researchers in SMI centers and regional tourism.

These collaborative structures are crucial for enhancing the quality and practical relevance of research findings, particularly within the context of globalization and complex local economic challenges. The international partnerships embedded within this network facilitate the transfer of knowledge and technological innovation, enabling effective implementation across SMI centers and regional tourism destinations. Ultimately, this authorship network analysis confirms that leading scholars and institutions are actively advancing the field, establishing a empirical foundation for future policy development and sustainable innovation strategies.

The visualization in Figure 4 illustrates the author citation network, which is highly informative for identifying intellectual influence patterns within strategic innovation and regional tourism. Several authors—such as Boes K., Buhalis D., and Inversini A.—appear as central nodes with large node sizes, indicating high citation counts and substantial scholarly impact. The strong ties among these researchers highlight their foundational contributions to the theoretical and empirical structure of the field. For instance, Buhalis D. is widely recognized for advancing the concepts of smart tourism and destination digitalization, which closely align with innovation strategies in SMI centers. Additionally, other prominent scholars like Pulido-Fernández J.I. and Rodríguez occupy strategic bridging positions, demonstrating their role in expanding cross-domain research perspectives.

**Figure 4.**
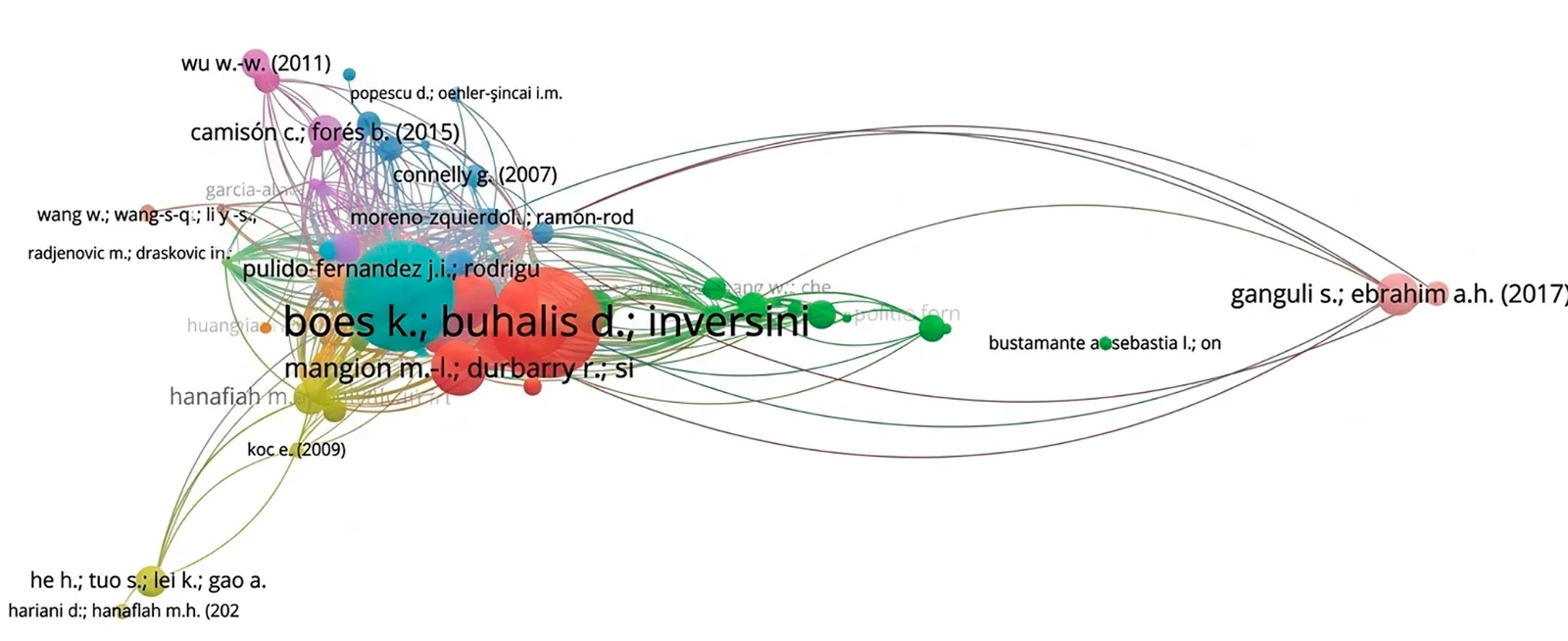
Author citation network visualization in strategic innovation research.

Interestingly, more peripheral nodes—such as those highlighted in niche domain studies—represent specialized subtopics that, despite lower overall node connectivity, contribute uniquely to the broader intellectual discourse. Beyond highlighting key actors, this citation network illustrates how strategic innovation concepts evolve and transform through scholarly dialogue. Overall, this citation analysis confirms that research on strategic innovation in SMI centers and regional tourism is backed by a robust intellectual structure, providing a clear roadmap for future interdisciplinary collaborations.

The bibliometric mapping results in Figure 4 align with the theoretical evolution previously synthesized in Table 2. The visualization shows a clear transition across research clusters, where early foundational nodes focus on industrial efficiency, whereas more recent clusters are dominated by terms such as “Strategic Innovation” and “Sustainable Tourism.” This outcome validates the conceptual shift from a pre-pandemic operational focus to innovation-driven recovery patterns. Furthermore, Figure 5 illustrates the collaboration network among institutions active in strategic innovation and regional tourism research. As shown in the overlay visualization, institutional nodes span global universities and research centers, with colors representing publication timelines from 2018 to 2024. Yellow nodes highlight newly active institutions, signaling an accelerating research investment in SMI-based tourism innovation in recent years.

**Figure 5.**
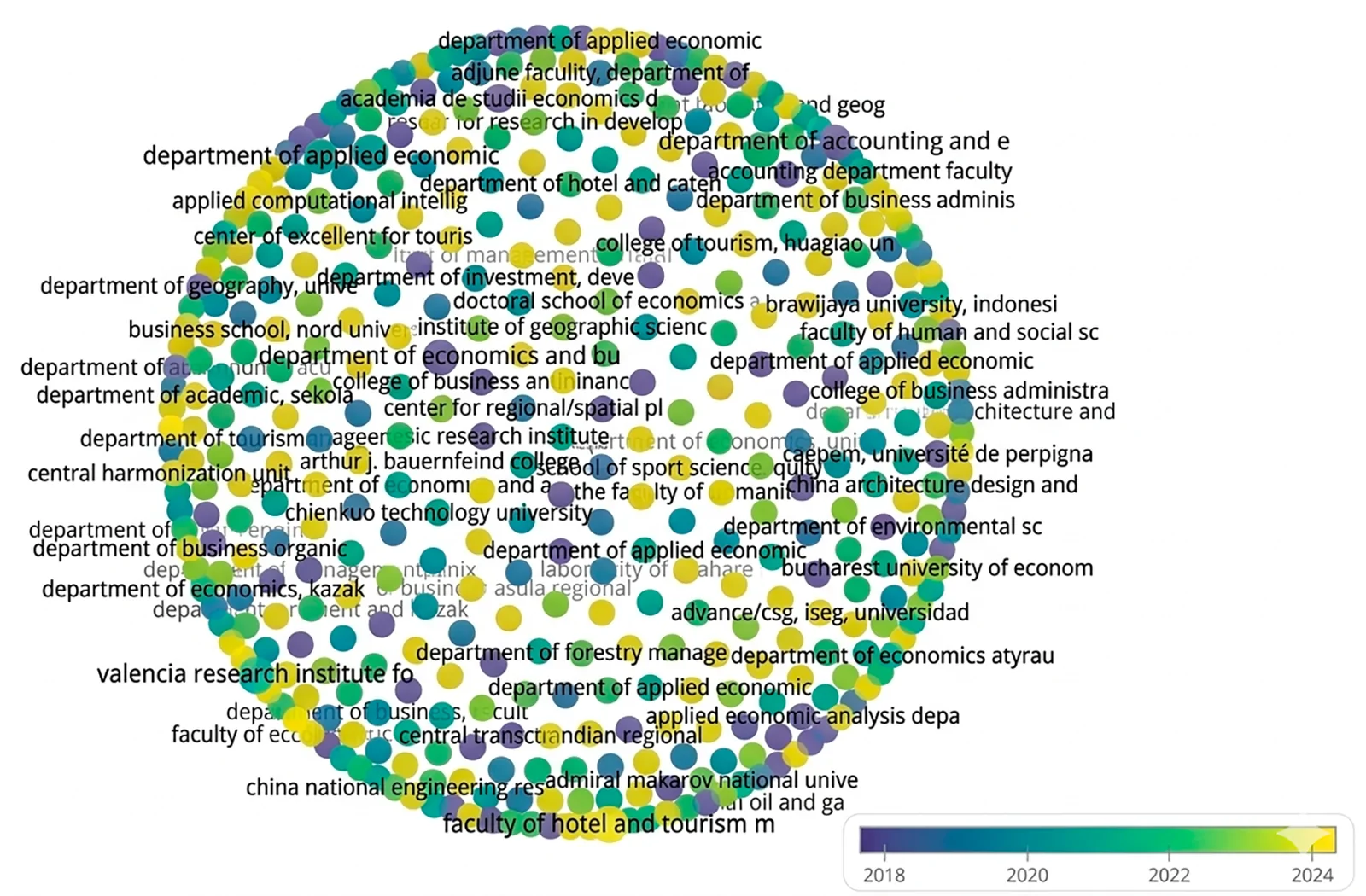
Overlay visualization of institutional collaboration networks contributing to SMI centers and regional tourism research.

Prominent institutions—such as Brawijaya University (Indonesia), China National Engineering Research Center, Chienkuo Technology University, and Valencia Research Institute—play pivotal roles in this collaboration network. The presence of these key entities underscores that strategic innovation within regional SMI centers and tourism is a global concern with strong cross-border relevance. Furthermore, the network exhibits multidisciplinary integration across faculties, spanning economics, business, tourism, engineering, and social sciences, which is essential for tackling complex sustainable development challenges.

Overall, this overlay visualization confirms that research on strategic innovation in SMI centers and regional tourism is sustained by a broad institutional ecosystem. This structural diversity enhances academic quality and accelerates knowledge transfer across borders. By establishing leading universities and centers as innovation hubs, these global networks provide the necessary foundation for strengthening the competitiveness and resilience of regional SMIs through evidence-based policies.

The bibliometric clusters highlighting “Competitiveness” and “Service” underscore the fundamental role of operational excellence in regional industry hubs. This aligns with empirical findings by prior studies, ^25^ which demonstrated that product and service quality serve as primary drivers of customer satisfaction in regional business clusters, such as those in Bogor. Therefore, continuous performance improvements are essential for SMIs to maintain their integration within regional tourism ecosystems.

As illustrated in Figure 6, the institutional network highlights focused inter-organizational linkages across key research groups. The cluster on the left represents core research centers and universities active in economics, tourism, and innovation management. Strong inter-institutional linkages among these specialized entities confirm that multidisciplinary collaboration is a defining characteristic of this domain. This integration is critical, as strategic innovation within SMI and tourism centers requires a holistic approach combining economic strategy, operations management, and destination development.

**Figure 6.**
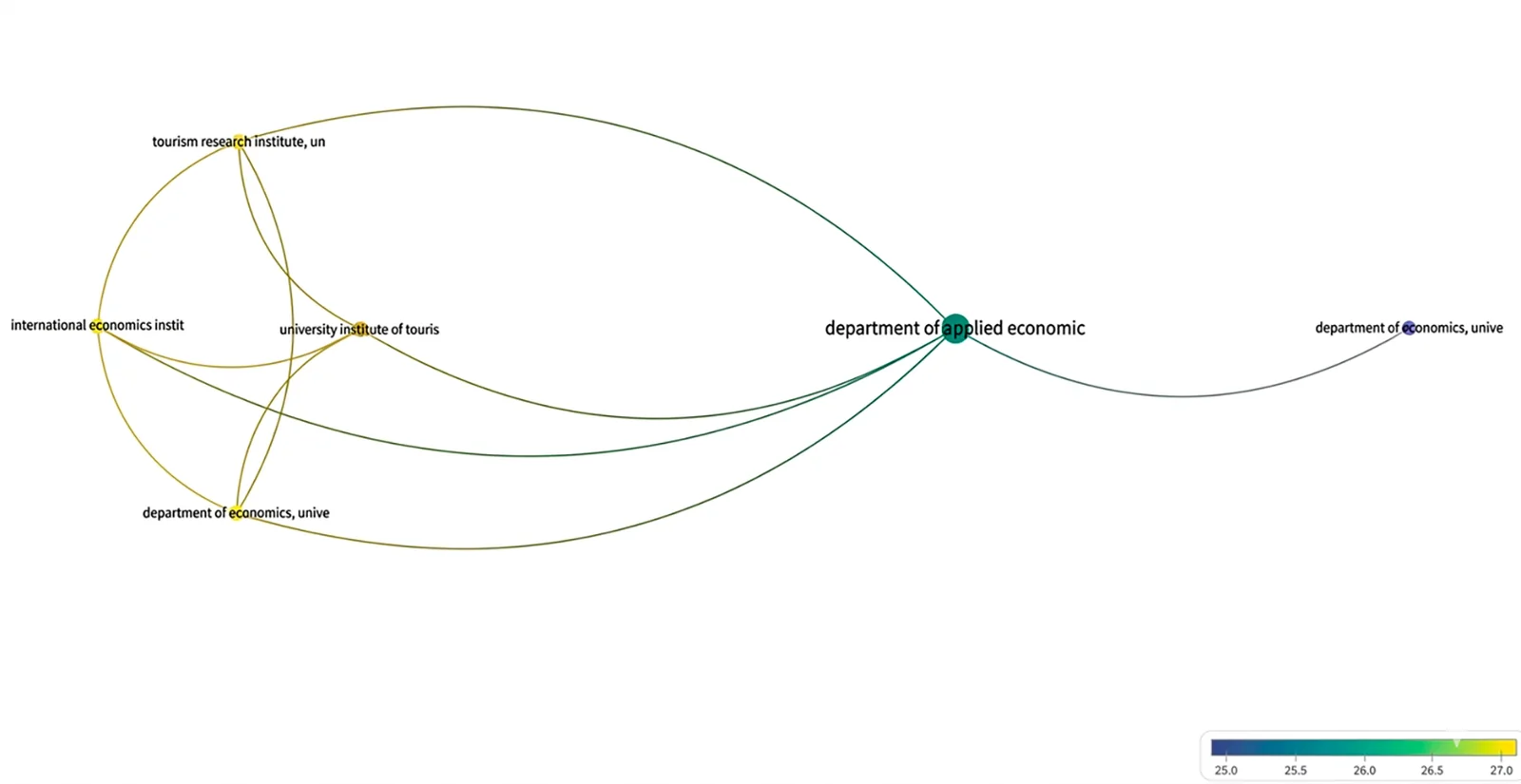
Inter-institutional collaboration network in strategic innovation and tourism research.

The Department of Applied Economics occupies a central bridging position within this network, connecting the primary institutional cluster on the left with more isolated nodes on the right. This strategic location highlights its pivotal role in facilitating cross-group knowledge transfer and expanding academic research networks. Although certain nodes display lower connectivity, the overall network demonstrates a cohesive collaborative structure. Such inter-institutional partnerships enhance research capacity and foster synergies critical for implementing sustainable innovation strategies in regional SMI centers.

Furthermore, bibliometric findings indicate that strategic positioning and internal capability are central nodes in current research trends. This aligns with foundational research frameworks, ^26^ which emphasize that evaluating internal and external business environments is crucial for small enterprises to leverage location-specific advantages and market dynamics. By systematically identifying these factors, SMI clusters can better align operational capabilities with regional tourism demands. Ultimately, this visualization confirms that a multidisciplinary institutional network underpins strategic innovation in regional SMIs, providing a key asset for addressing complex economic challenges and optimizing academic resources.

The analysis of the cross-country collaboration network, as summarized in Figure 7, illustrates international research partnerships in strategic innovation within SMI centers and regional tourism. The visualization depicts a robust and diverse network, demonstrating that this domain holds significant global relevance. Countries such as Spain, China, Indonesia, and Portugal serve as major collaborative hubs, as indicated by their large node sizes and extensive degree centrality. This structural prominence indicates that these nations not only generate high publication volumes but also lead international research networks that elevate overall scholarly impact.

**Figure 7.**
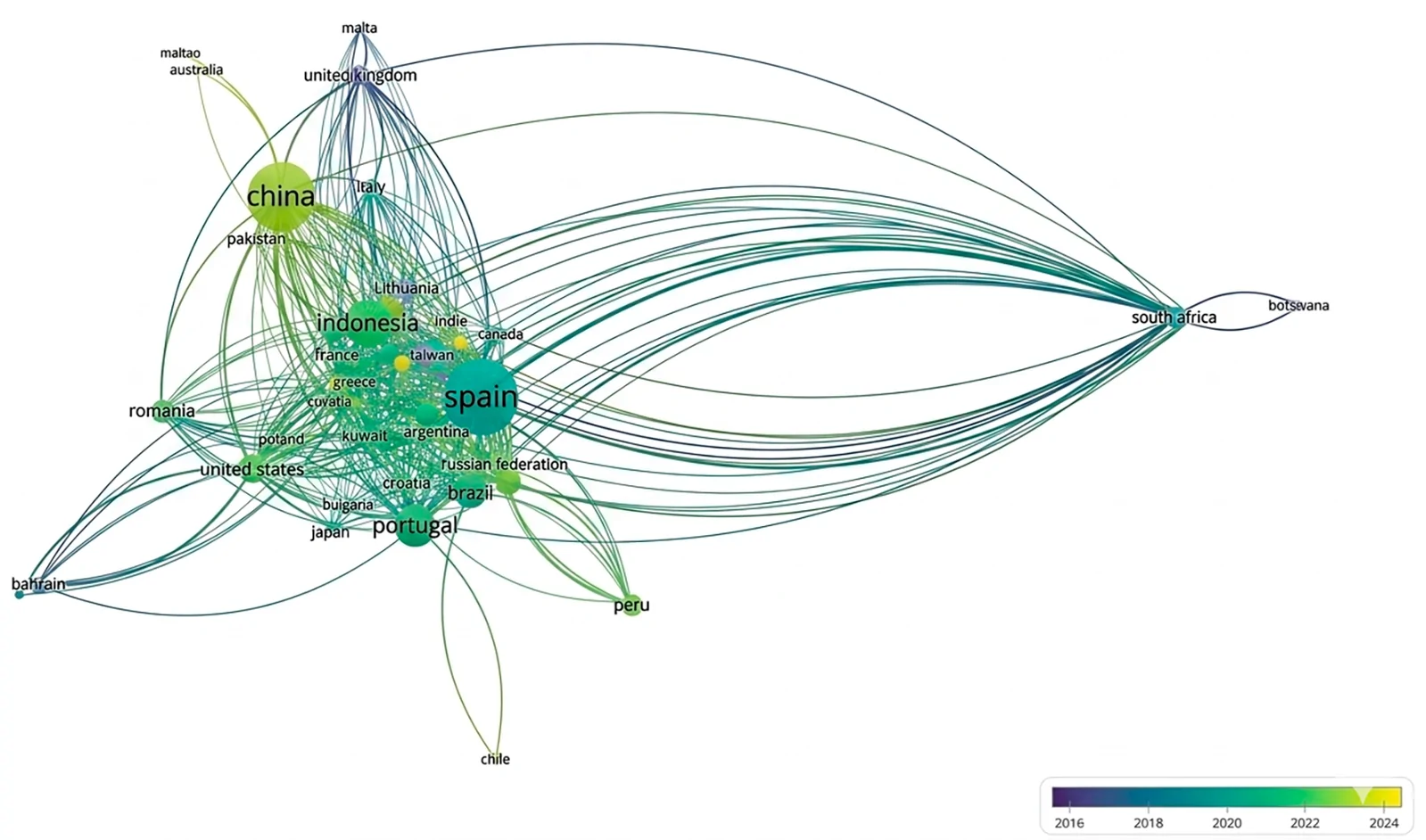
International collaboration network of active countries in SMI centers and regional tourism research.

Notably, Indonesia emerges as a key node within this global network, reflecting its active engagement in international research initiatives. This integration emphasizes that strategic innovation within Indonesian SMI centers and regional tourism development benefits from continuous knowledge exchange with the broader academic community. Furthermore, connections extending to countries across Latin America (e.g., Brazil, Peru), Africa (e.g., South Africa), and Europe highlight a truly cross-continental and multidisciplinary research structure. Such global collaboration is vital for enriching research perspectives and adapting strategic innovations to diverse socio-economic contexts.

Node colors ranging across publication years (2016 to 2024) illustrate dynamic trends and intensifying collaboration in recent years. This trajectory indicates that research in this domain is experiencing sustained growth while remaining adaptive to evolving global challenges. Overall, the international collaboration network reflects a robust, mutually supportive research ecosystem—a fundamental requirement for advancing strategic innovation within regional SMI centers. Furthermore, global partnerships elevate academic rigor and accelerate the cross-border transfer of best practices, ultimate strengthening local tourism competitiveness in a sustainable manner.

### Dominant keywords and central themes in strategic innovation and regional tourism research

As illustrated in Figure 8, the keyword co-occurrence network highlights the dominant themes and conceptual structures within strategic innovation research. The keyword “competitiveness” emerges as the central node with the highest degree centrality and node size, confirming that enhancing competitive advantage is the primary focus of this literature body. Surrounding this central node are closely interconnected clusters covering keywords such as “innovation,” “tourist destination,” “sustainable development,” and “tourism industry.” This network topology demonstrates that innovation is conceptualized not merely as an operational tool, but as a strategic driver for long-term destination sustainability and regional growth.

**Figure 8.**
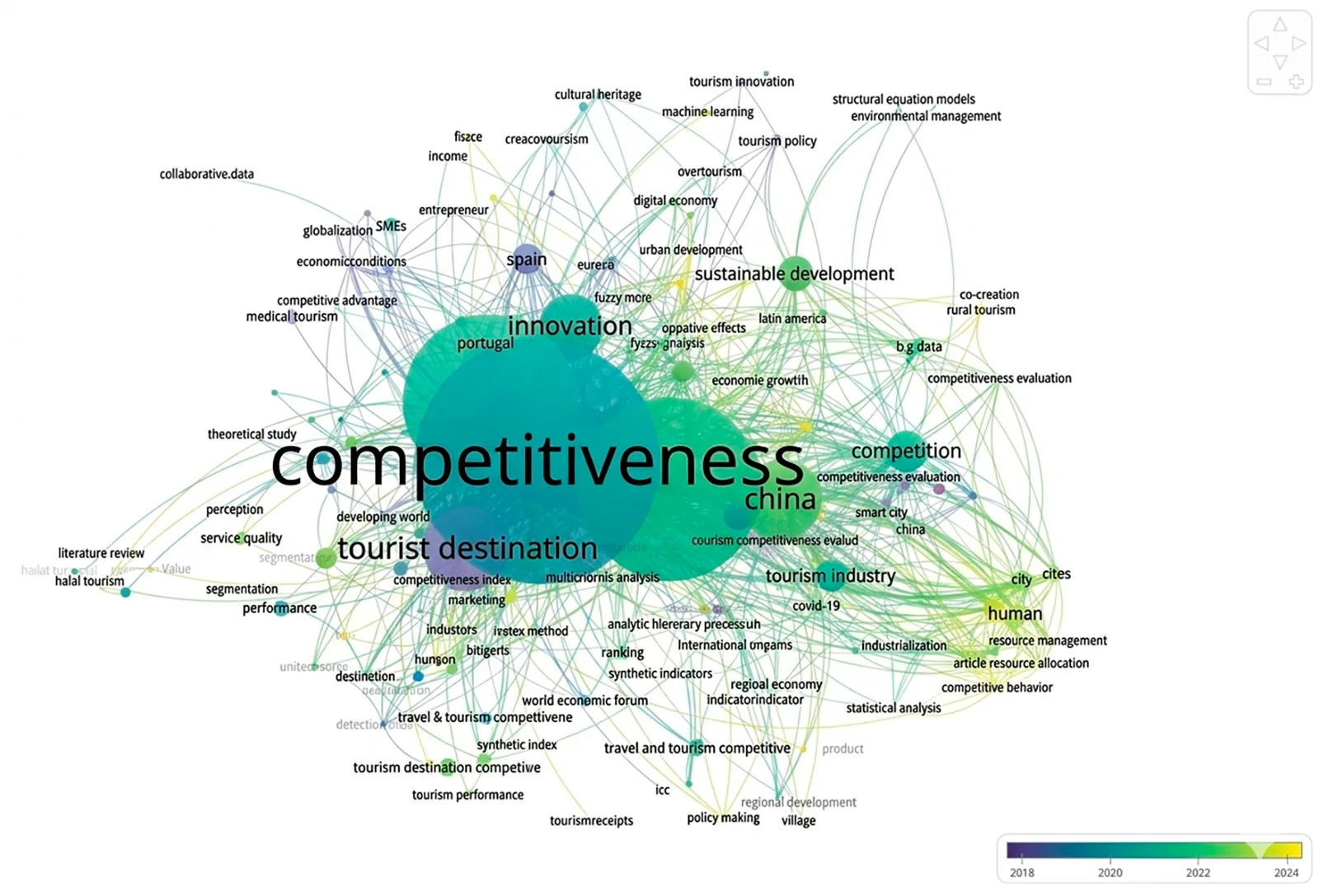
Keyword co-occurrence network visualization in strategic innovation and tourism literature.

The prominent “sustainable development” cluster underscores that environmental and social responsibility has become a central focus in recent literature. This domain closely links with concepts such as “environmental management” and “co-creation,” signaling a shift toward collaborative resource management within innovation frameworks. Furthermore, emerging keywords such as “SMIs,” “digital economy,” “big data,” and “machine learning” reflect the rapid integration of digital technologies into tourism innovation. These technical capabilities serve as key drivers for operational efficiency and personalized visitor experiences, highlighting the imperative for SMI centers to embrace digital transformation to sustain product and service competitiveness.

The “tourist destination” and “tourism industry” clusters emphasize that scholarly focus extends beyond product-level innovations to encompass broader destination management dynamics. Strong linkages with terms such as “policy making,” “regional development,” and “competitive behavior” demonstrate that market policies and strategic behavior are integral components of effective innovation frameworks. Overall, this keyword network depicts a multidisciplinary research landscape that integrates economic, technological, environmental, and policy perspectives to enhance regional tourism competitiveness. Ultimately, this visualization illustrates how interconnected concepts form a cohesive theoretical framework in the field.

### Shared citation patterns and bibliographic coupling networks in strategic innovation research

The bibliographic mapping in Figure 9 illustrates the cumulative growth and evolutionary trajectories of primary keywords over the past two decades. Notably, the term “competitiveness” occupies the most dominant position, exhibiting sharp cumulative growth—particularly since 2017—which confirms it as the core thematic driver in SMI and regional tourism research. Similarly, terms such as “tourism” and “tourism competitiveness” show strong upward momentum, emphasizing the strategic role of destination competitiveness within regional economies. Furthermore, the steady growth of “innovation” and “sustainability” demonstrates that innovation is increasingly viewed as an essential driver for sustainable development, aligning with global priorities to balance economic growth with environmental and social responsibility.

**Figure 9.**
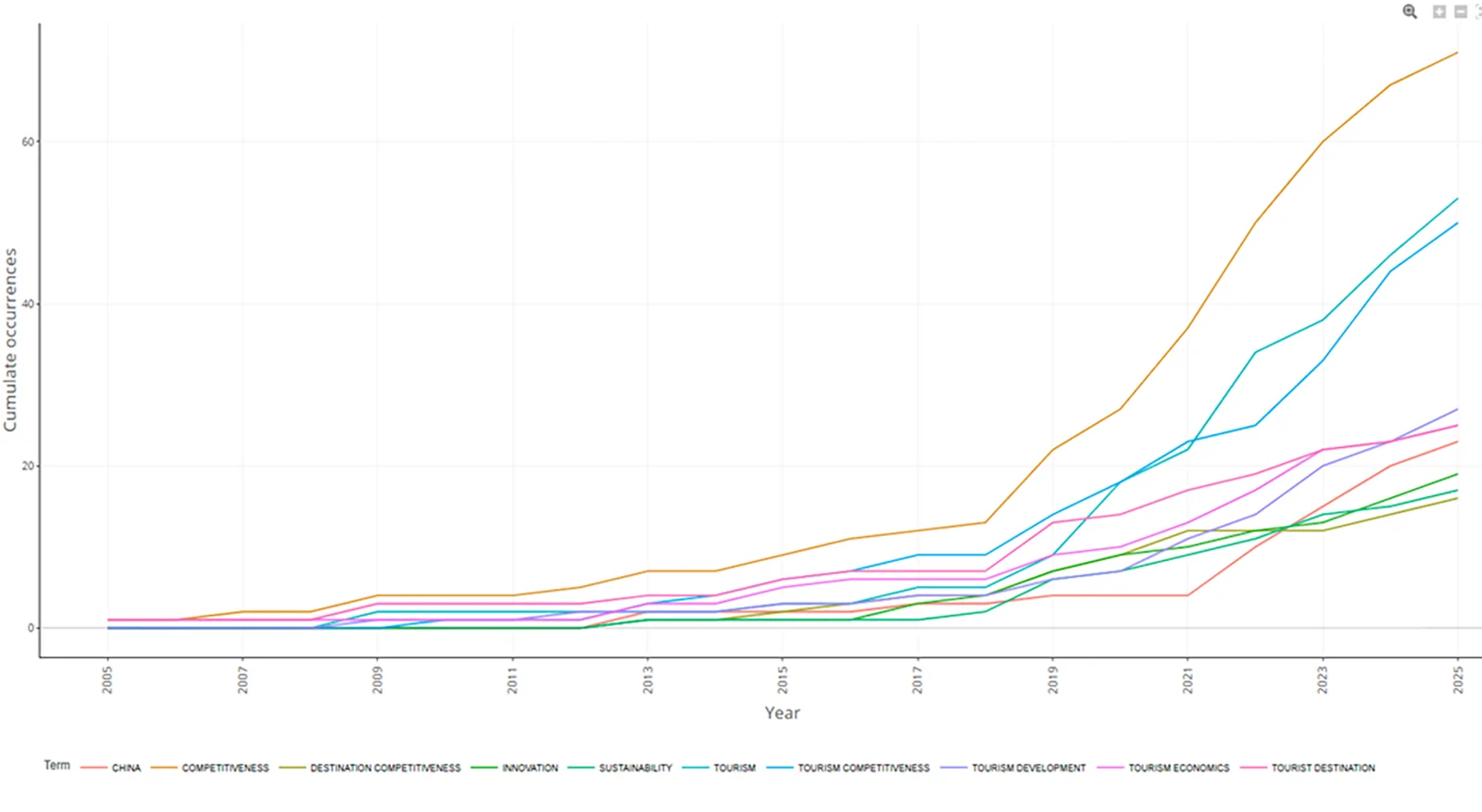
Cumulative growth trajectory of top keywords in strategic innovation and tourism research (2005–2025).

To further examine these interconnections, Figure 9 illustrates the bibliographic network mapping the relationships between documents, leading authors, contributing institutions, and representative countries. The network visualization highlights distinct clusters of collaboration, where key global institutions and prolific authors act as knowledge hubs, driving cross-border research integration. Countries with well-established academic infrastructure exhibit dense co-authorship ties, signaling strong international partnerships in strategic innovation within tourism and regional SMIs. Ultimately, this structural network underscores how collaborative knowledge-sharing accelerates the dissemination and global impact of regional tourism management strategies.

The structural mapping in Figure 10 illustrates the publication and collaboration patterns across corresponding authors’ countries in strategic innovation research. China dominates total publication volume, followed closely by Spain and Indonesia. This highlights the central role of these three nations in advancing research within SMI centers and regional tourism development.

**Figure 10.**
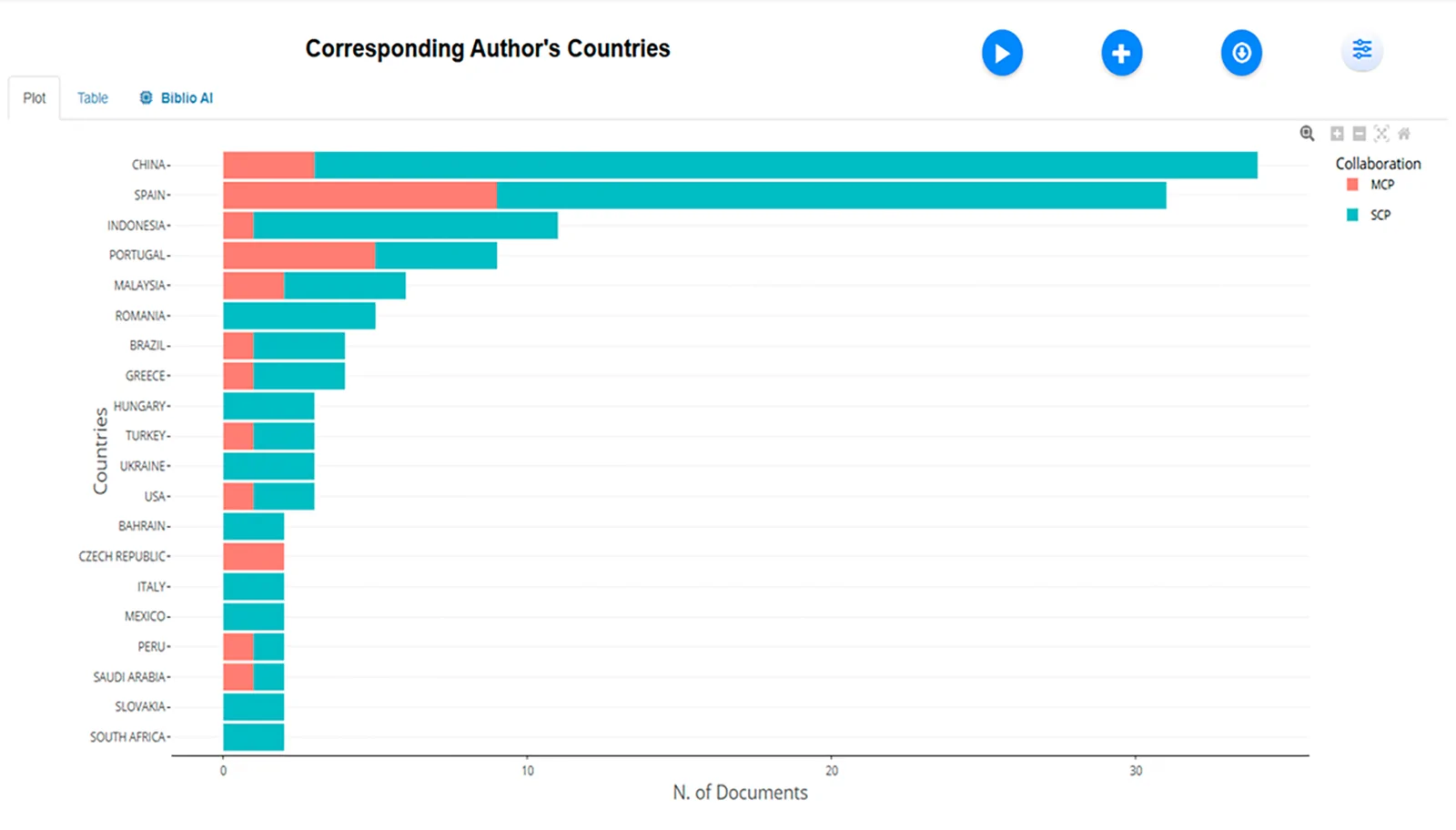
Corresponding author countries and international collaboration ratios in strategic innovation and tourism research.

China’s significant contribution is evident in its substantial volume of Single Country Publications (SCP) alongside a notable proportion of Multiple Country Publications (MCP). This demonstrates that research from China possesses both strong domestic capacity and active international collaboration networks that expand its global reach. Spain ranks second, characterized by a high proportion of MCPs, which reflects an extensive research network and deep integration within the global academic community. Importantly, Indonesia displays a strong publication presence with an emphasis on international collaborations, signaling openness and active participation in global research partnerships within strategic innovation and tourism.

Other countries, such as Portugal, Malaysia, and Romania, also show a growing research presence. This geographical distribution highlights the expanding global attention toward developing SMI centers and destination competitiveness across diverse regional contexts. The variance between MCPs and SCPs reflects differing levels of international engagement: countries with high MCP proportions demonstrate strong cross-border collaborative capacity, which is critical for elevating research quality and impact. Conversely, SCP-dominated literature indicates a stronger focus on national agendas, which can be further encouraged toward global partnerships. Ultimately, international collaboration accelerates knowledge transfer, strengthening long-term innovation and competitiveness in regional tourism ecosystems.

The structural dynamics presented in Figure 11 illustrate the evolution and interconnectedness among documents, authors, institutions, and keywords in strategic innovation research.

**Figure 11.**
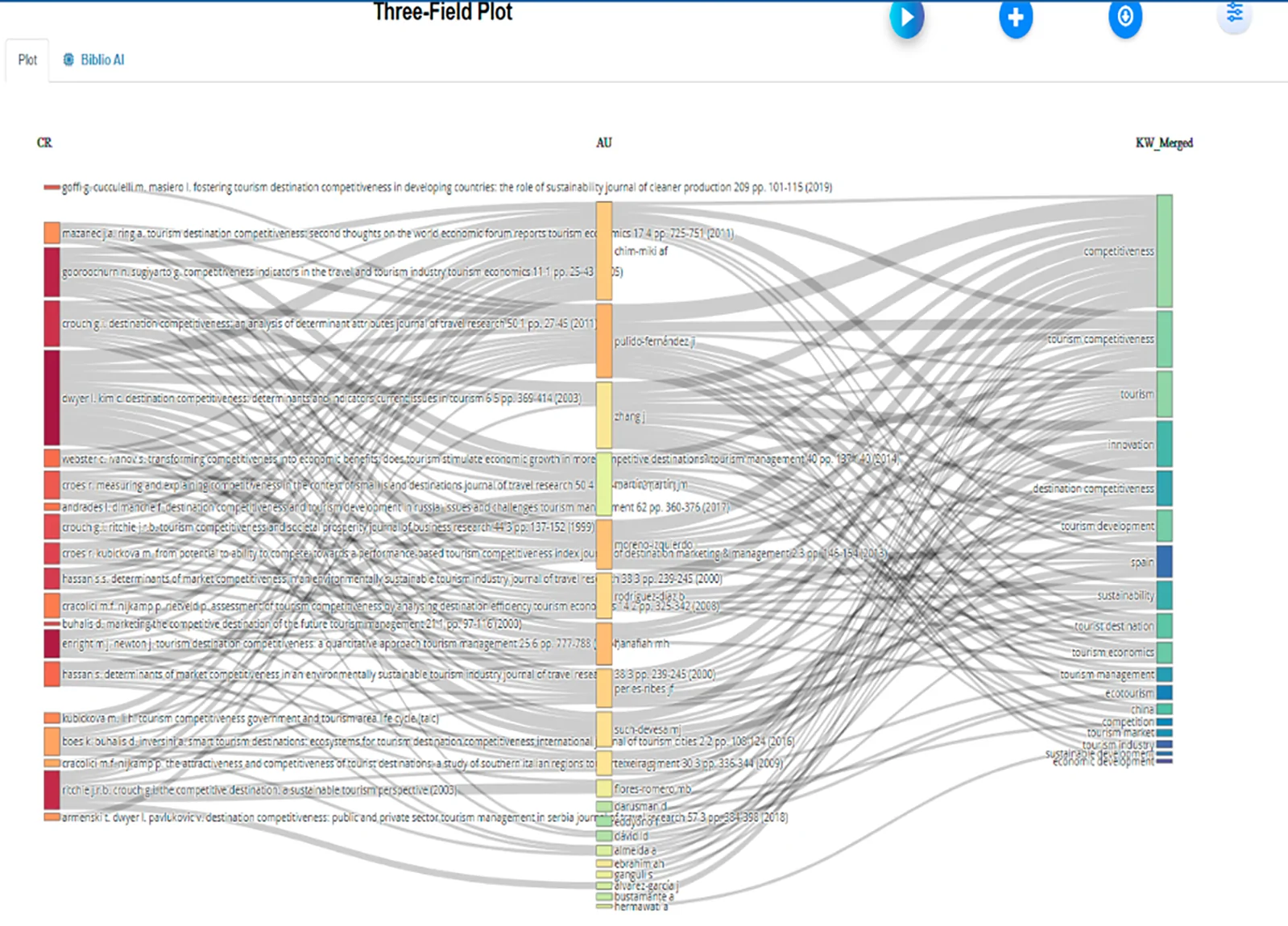
Three-fields plot illustrating connections among cited documents (left), authors (middle), and keywords (right) in strategic innovation research.

On the left column, the most-cited documents provide foundational theoretical and empirical frameworks, including seminal works such as those by reference 27, which examines sustainability in enhancing destination competitiveness in developing nations, and reference 28, which evaluates destination competitiveness relative to World Economic Forum standards. These core documents serve as intellectual anchor points shaping current research directions. In the center, prominent scholars such as Pulido-Fernández J.I., Zhang J., and Buhalis D. act as key knowledge bridges, connecting foundational literature to evolving research themes. Their central positioning in the network highlights their influential role in developing strategic innovation models that are directly applicable to SMIs and tourism development.

On the right column, the dominant keywords include “competitiveness,” “tourism competitiveness,” and “tourism,” confirming that enhancing destination performance through strategic innovation remains the primary research focus. Additionally, keywords such as “innovation,” “destination competitiveness,” “sustainability,” and “tourism development” establish innovation and sustainability as essential strategic pillars. The network demonstrates strong linkages between sustainability themes and competitive innovation, reflecting a growing trend toward integrating environmental and social considerations into SMI-based tourism development strategies. Overall, this multi-field analysis reveals how the strategic innovation discourse in regional SMI centers is anchored in robust foundational literature, guided by influential authors, and focused on timely, relevant themes. Ultimately, this visualization underscores the necessity of harmonizing competitiveness, innovation, and sustainability to shape resilient regional tourism practices.

The thematic evolution and projection map presented in Figure 12 illustrates the historical shifts in research focus from 2005 to 2024 alongside projected directions for 2025. The evolutionary pathways demonstrate that core themes, particularly “tourism competitiveness,” maintain a central trajectory and are expected to continue dominating the literature. This ongoing dominance confirms that enhancing destination performance remains a enduring priority for both academic scholars and industry practitioners.

**Figure 12.**
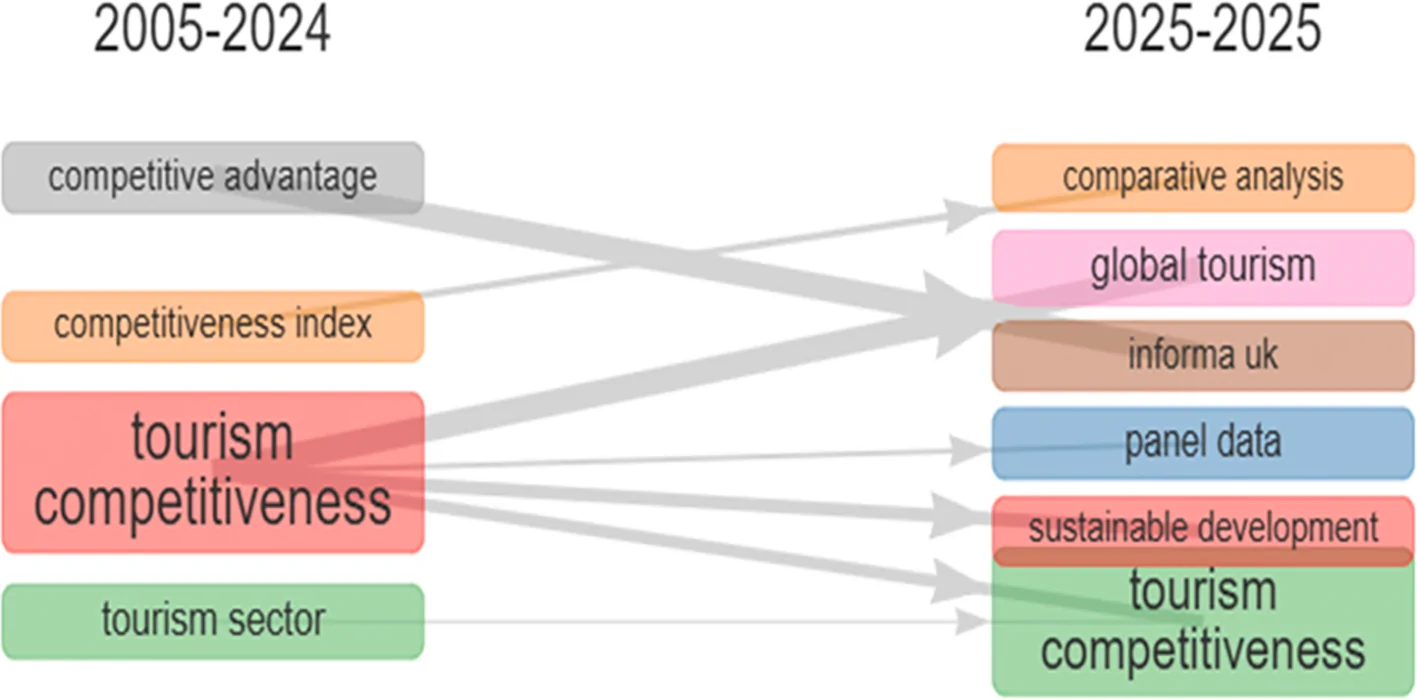
Thematic evolution and projection of strategic innovation research in SMI centers for regional tourism competitiveness.

Additionally, foundational themes such as “competitive advantage” and “competitiveness index” have shifted toward more quantitative and comparative analytical frameworks. Emerging focus areas like “comparative analysis” and “panel data” indicate growing methodological rigor through longitudinal datasets and cross-contextual assessments. Simultaneously, projections for “global tourism” suggest an expanding international scope, reflecting the cross-border nature of modern tourism markets.

Furthermore, “sustainable development” has integrated deeply into the core trajectory, signaling a paradigm shift toward growth strategies that balance economic efficiency with environmental and social sustainability. ^29^ ^,^ ^30^ Overall, these thematic shifts demonstrate that tourism competitiveness research has evolved from basic measurement indices to a multidimensional, globally conscious, and methodologically advanced domain equipped to address complex future challenges.

### Contributions and practical implications for strategic innovation in SMI centers and regional tourism competitiveness

The bibliometric analysis reveals a steady growth in publications focused on strategic innovation within Small and Medium Industry (SMI) centers to enhance regional tourism competitiveness since 2010. Dominant thematic streams center around digitalization, sustainability, innovative product development, and multi-stakeholder collaboration. Geographically, major contributions emerge from Southeast Asia—particularly Indonesia—alongside expanding international research networks. Primary keyword hubs such as “digital innovation,” “sustainable development,” and “tourism competitiveness” reflect a progressive consolidation of knowledge in this field. By mapping these two decades of literature, this study provides actionable insights for scholars and policymakers seeking to implement strategic innovation frameworks in regional SMI hubs.

The primary contribution of this research lies in identifying the convergence of digitalization and sustainability as the core engine of strategic innovation in SMI centers to boost destination competitiveness. These findings confirm that innovation extends beyond product-specific or technical enhancements to encompass business model transformation, adaptive resource management, and cross-sectoral collaboration capable of responding to market and environmental dynamics. ^31^ ^–^ ^36^ From a practical standpoint, these insights offer a robust empirical foundation for policymakers, SMI operators, and regional tourism stakeholders to formulate structured innovation roadmaps. ^37^ ^–^ ^41^ Prioritizing multi-stakeholder governance and digital capacity building serves as a key strategic lever to accelerate technology adoption and sustainable practices at the local level. ^29^ ^,^ ^42^ ^–^ ^45^

Additionally, this research establishes a framework for evidence-based and contextual innovation models that enhance both SMI economic performance and the broader regional tourism ecosystem. ^30^ ^,^ ^46^ These implications are particularly vital in addressing challenges related to globalization, evolving tourist behavior, and social-environmental sustainability. Ultimately, this study contributes theoretically by enriching the literature on strategic innovation while delivering practical impact by strengthening SMI centers to drive destination competitiveness. ^47^ ^–^ ^51^ Looking ahead, these findings offer a baseline for future empirical investigations and adaptive, context-specific innovation policies.

In summary, this study affirms that integrating digitalization and sustainability serves as the primary engine for increasing tourism competitiveness anchored in SMI centers. ^52^ ^–^ ^54^ Expanding publication volumes and cross-border collaborations reflect a growing consensus on the need for holistic innovation approaches. However, cross-country and cross-sectoral partnerships must be further leveraged to accelerate knowledge transfer and capacity building. ^55^ Practically, policies must prioritize digital enablement for SMIs, continuous skills development, and the creation of an inclusive innovation ecosystem. Future research should explore empirical execution across diverse destinations to evaluate direct impacts on local economic growth.

These empirical findings affirm that strategic innovations integrating digitalization and sustainability serve as the primary drivers of tourism competitiveness in Small and Medium Industry (SMI) centers. Digitalization not only enhances operational efficiency but also expands market reach and enriches overall traveler experiences. ^6^ ^,^ ^17^ Furthermore, embedding sustainability principles ensures that regional tourism development safeguards natural ecosystems and supports local community well-being. ^7^ ^,^ ^22^ ^,^ ^56^ ^,^ ^57^

The growing volume of publications and cross-institutional collaborations reflects an increasing consensus on the necessity of holistic, multidisciplinary approaches. Studies such as. ^24^ ^,^ ^58^ ^–^ ^60^ emphasize that multi-stakeholder collaboration and co-creation form the cornerstone of sustainable, market-responsive innovation. However, cross-border and cross-sectoral synergies remain relatively constrained, posing an obstacle to global knowledge transfer and capacity building. ^4^ ^,^ ^5^ Consequently, expanding international and cross-sectoral networks represents a strategic priority to accelerate innovation diffusion. The synergy between strategic innovation and cluster development acts as a core engine of regional growth. As demonstrated in Ref. 61, entrepreneurial activities within targeted industrial and agricultural sectors substantially drive economic performance in Indonesia, offering a clear model for how SMI clusters can invigorate local economies and support long-term regional stability.

From a policy perspective, these findings emphasize the necessity of regulatory frameworks and targeted programs that promote SMI digitalization, provide access to technology, offer workforce training, and incentivize sustainable innovation. ^3^ ^,^ ^8^ ^,^ ^12^ ^,^ ^62^ ^–^ ^64^ Establishing an inclusive, adaptive innovation ecosystem requires active synergies among government entities, the private sector, academia, and local communities. ^10^ ^–^ ^12^ ^,^ ^65^ Furthermore, future empirical studies should evaluate field-level implementations to measure direct impacts on local economies and destination performance. Such context-specific empirical validation is crucial to refine innovation models tailored to SMI centers. ^21^ ^,^ ^66^ ^–^ ^70^ Overall, these insights reinforce that combining digitalization and sustainability is key to transforming SMI centers into core drivers of regional tourism competitiveness through inclusive and sustainable economic development. ^71^

Additionally, establishing a distinct brand identity is essential for SMIs within regional tourism clusters. As highlighted in Ref. 72, brand experience and customer satisfaction play pivotal roles in cultivating brand loyalty, mediated by consumer trust. Integrating strategic branding elements into SMI innovation frameworks enhances market recognition and secures long-term competitiveness for tourism-based clusters.

## Conclusion

This bibliometric study successfully maps the evolution of strategic innovation trends within Small and Medium Industry (SMI) centers and their impact on regional tourism competitiveness over the past two decades. The findings confirm that digitalization and environmental sustainability serve as the twin fundamental pillars driving modern strategic innovation, demonstrating that competitiveness transcends mere technological adoption to encompass inclusive, resilient economic development. Strategically, fostering multi-stakeholder collaboration—encompassing government, industry, academia, and local communities—is essential for accelerating adaptive innovations that respond to evolving market and environmental demands. Ultimately, future research should build upon these insights through empirical field implementations to quantitatively evaluate direct impacts on local economic performance and destination resilience.

In the future, further research should empirically examine the implementation of these strategic innovations to evaluate their direct impacts on local economic growth and tourism sustainability. Such empirical work will enrich practical understanding while establishing a robust scientific foundation for more effective innovation policies. Consequently, this study delivers a significant theoretical contribution and provides a practical framework for strengthening SMI centers as catalysts for sustainable, highly competitive regional tourism.

## Ethical considerations

As this study relies exclusively on bibliometric analysis of previously published academic literature, it did not involve human participants, clinical trials, or animal experimentation. Therefore, formal ethical approval was not required. Furthermore, the authors declare that this research entails no negative societal, ethical, or environmental impacts.

## Data Availability

The underlying data supporting this study is openly available on the Open Science Framework under the title “Strategic Innovation Trends in Small and Medium Industry Clusters for Enhancing Regional Tourism Competitiveness: A Bibliometric Analysis” at https://doi.org/10.17605/OSF.IO/8PMZK. ^73^ This repository contains the raw metadata extracted from the Scopus database (scopus_metadata.csv), which includes all retrieved documents used for the bibliometric and systematic analysis. Data are available under the terms of the Creative Commons Zero v1.0 Universal (CC0 1.0) Public Domain Dedication. OSF: Strategic Innovation Trends in Small and Medium Industry Clusters for Enhancing Regional Tourism Competitiveness: A Bibliometric Analysis. https://doi.org/10.17605/OSF.IO/8PMZK. ^73^ This repository contains the high-resolution VOSviewer network visualization maps and supplementary search string details. Data are available under the terms of the Creative Commons Zero v1.0 Universal (CC0 1.0) Public Domain Dedication. OSF: PRISMA 2020 Checklist for “Strategic Innovation Trends in Small and Medium Industry Clusters for Enhancing Regional Tourism Competitiveness: A Bibliometric Analysis”. https://doi.org/10.17605/OSF.IO/8PMZK. ^73^ Data are available under the terms of the Creative Commons Zero v1.0 Universal (CC0 1.0) Public Domain Dedication.
