# Supplementary material for: Strategic Innovation Trends in Small and Medium Industry Clusters for Enhancing Regional Tourism Competitiveness: A Bibliometric Analysis
Source: F1000Res. 2026 Oct 9;15:1006. Originally published 2026 Jun 24. [Version 2] doi: 10.12688/f1000research.183398.2 (PMC13728438; doi:10.12688/f1000research.183398.2)
Supplement: Peer review history

# Reviewer report: Reviewer response for version 2

**DOI:** 10.5256/f1000research.209211.r528505

**Published:** 2026-11-02

**Authors:** Fandi Achmad

**Affiliations:** 1 Telkom University, Bandung, Indonesia

The revised manuscript has demonstrated substantial improvement in response to the comments and recommendations provided during the first round of peer review.

 The authors have made meaningful revisions to improve the clarity, coherence, and academic presentation of the manuscript. The revised version presents a more focused discussion of strategic innovation, digitalization, sustainability, and regional tourism competitiveness. The overall structure and narrative flow have also been improved, making the research findings and their implications easier to follow.

 The revisions have satisfactorily addressed my previous concerns, and I consider the manuscript to have reached an acceptable scientific standard for indexation. The study provides a relevant contribution to the literature on strategic innovation and regional tourism development through its bibliometric analysis. I have no further substantive comments or recommendations for revision.

Is the work clearly and accurately presented and does it cite the current literature?

Partly

If applicable, is the statistical analysis and its interpretation appropriate?

Partly

Are all the source data underlying the results available to ensure full reproducibility?

Partly

Is the study design appropriate and is the work technically sound?

Partly

Are the conclusions drawn adequately supported by the results?

Partly

Are sufficient details of methods and analysis provided to allow replication by others?

No

Reviewer Expertise:

Sustainable Performance, Sustainability, Organizational Development, Innovation Management

I confirm that I have read this submission and believe that I have an appropriate level of expertise to confirm that it is of an acceptable scientific standard.

# Reviewer report: Reviewer response for version 2

**DOI:** 10.5256/f1000research.209211.r528507

**Published:** 2026-10-19

**Authors:** Anas Pattaray, Endang Sri Wahyuni, Lalu Ratmaja

**Affiliations:** 1 Tour and Travel Business Management Study Program, Politeknik Pariwisata Lombok, Central Lombok Regency, West Nusa Tenggara, Indonesia; 2 Tour and Travel Business Management, Politeknik Pariwisata Lombok, Central Lombok Regency, West Nusa Tenggara, Indonesia

Thank you to the authors. You have incorporated several of my corrections. I have reviewed the sections I corrected. I believe this article is worthy of indexing. I am confident that this article will contribute to scientific literacy and serve as a foundation for future research.

Is the work clearly and accurately presented and does it cite the current literature?

Partly

If applicable, is the statistical analysis and its interpretation appropriate?

Partly

Are all the source data underlying the results available to ensure full reproducibility?

Partly

Is the study design appropriate and is the work technically sound?

Partly

Are the conclusions drawn adequately supported by the results?

Partly

Are sufficient details of methods and analysis provided to allow replication by others?

Partly

Reviewer Expertise:

• Sustainable Tourism Development • Halal and Muslim-Friendly Tourism • Destination Planning and Governance • Community-Based and Rural Tourism • Tourism and Cultural Heritage • Disability and Accessible Tourism • Tourism Human Resource Development

We confirm that we have read this submission and believe that we have an appropriate level of expertise to confirm that it is of an acceptable scientific standard.

# Reviewer report: Reviewer response for version 1

**DOI:** 10.5256/f1000research.202442.r509921

**Published:** 2026-08-17

**Authors:** Fandi Achmad

**Affiliations:** 1 Telkom University, Bandung, Indonesia

The manuscript addresses a relevant and timely topic and provides a broad bibliometric mapping of strategic innovation, SMI clusters, and regional tourism competitiveness. The use of Scopus/Web of Science, VOSviewer, and Biblioshiny provides a suitable basis for the study. However, major revisions are required to ensure methodological consistency, reproducibility, and alignment between evidence and claims.

 Abstract

 #1. The claim that strategic innovation, digitalization, and collaboration act as "drivers" or directly enhance regional tourism competitiveness is too strong, given that this study relies solely on bibliometric analysis and does not test for causal relationships.

 Introduction

 #1. There is substantial repetition in the opening section regarding SMIs as the "backbone of the local economy," rendering the Introduction redundant and failing to build the argument efficiently.

 #2. The conceptual flow linking SMI clusters, strategic innovation, digitalization, sustainability, and regional tourism competitiveness lacks coherence; various concepts are introduced sequentially without a clear theoretical bridge.

 #3. The concept of regional tourism competitiveness is not explicitly defined; consequently, it remains unclear whether competitiveness refers to destination attractiveness, tourism performance, economic value, market competitiveness, or sustainability. I suggest consulting papers on competitiveness within the context of SMEs and tourism in Indonesia. DOI: https://doi.org/10.1016/j.joitmc.2025.100532 ; DOI: https://doi.org/10.1108/INMR-12-2023-0253

 #4. The research gap is framed too broadly merely stating that innovation and tourism have been studied "separately" without critically comparing previous bibliometric studies or identifying a specific knowledge gap.

 #5. There is an inconsistency between the Indonesian context heavily emphasized in the Introduction and the global nature of the bibliometric dataset; the study's positioning, whether as Indonesia-focused or global needs clarification.

 Materials and Methods

 #1. There is a serious inconsistency regarding database sources: the Methods section mentions Scopus in one part, while another part and Figure 1 state that both Scopus and Web of Science were used, making it impossible to verify the origin of the final 195 articles.

 #2. The document counts within the PRISMA flow are inconsistent; the Scopus/WoS figures in the Methods narrative differ from those in Figure 1, meaning the process from 3,295 records to 195 final documents cannot be fully audited or replicated.

 #3. The Boolean search string contains redundancies, such as "strategic innovation" and "competitiveness" and the inclusion of "small and medium enterprises" risks expanding the dataset well beyond the specific context of Small and Medium Industries.

 #4. The inclusion criterion of "open access/hybrid gold" risks introducing selection bias and lacks methodological justification as a standard for article quality.

 #5. The procedures for screening, deduplication, data cleaning, and exclusion lack sufficient transparency; details regarding the number of duplicates, reasons for full-text exclusion, deduplication software used, and metadata verification procedures are not provided.

 #6. The parameters for the bibliometric analysis are incomplete, missing details such as minimum occurrence/citation thresholds, keyword normalization, thesaurus cleaning, software versions (VOSviewer/Bibliometrix), counting parameters, and specific network analysis configurations - thereby limiting reproducibility.

 Results and Discussion

 #1. There appear to be inconsistencies in the bibliometric statistics presented in Figure 2, specifically regarding the relationship between the 521 authors, 195 documents, and the figure of 3.05 authors per document which require verification and explanation based on the indicator definitions used.

 #2. The Results section repeatedly interprets bibliometric associations as causal relationships; for instance, digitalization and sustainability are described as "drivers" of tourism competitiveness, whereas co-occurrence and citation analyses do not demonstrate causal effects.

 #3. There are discrepancies between the captions and the content of several figures, particularly Figures 9 and 10; Figure 9 actually depicts temporal keyword development, while Figure 10 shows corresponding-author countries (MCP-SCP) rather than a "bibliographic network between documents, authors, institutions, and countries."

 #4. Figure 12 mentions a "projection for 2025" but fails to explain the forecasting method employed; if the 2025 data represents actual figures, the term "projection" should be removed, as thematic evolution is not synonymous with forecasting.

 Conclusion

 #1. The Conclusion claims that the study maps "their impact on regional tourism competitiveness," whereas bibliometric analysis can only reveal research patterns, associations, and thematic evolution, rather than direct impacts or causal effects.

 #2. The most concrete bibliometric findings, such as the 195 publications, 15.86% annual growth rate, dominant competitiveness theme, thematic evolution, and the contributions from China, Spain, and Indonesia have not been explicitly summarized in the Conclusion.

Is the work clearly and accurately presented and does it cite the current literature?

Partly

If applicable, is the statistical analysis and its interpretation appropriate?

Partly

Are all the source data underlying the results available to ensure full reproducibility?

Partly

Is the study design appropriate and is the work technically sound?

Partly

Are the conclusions drawn adequately supported by the results?

Partly

Are sufficient details of methods and analysis provided to allow replication by others?

No

Reviewer Expertise:

Sustainable Performance, Sustainability, Organizational Development, Innovation Management

I confirm that I have read this submission and believe that I have an appropriate level of expertise to confirm that it is of an acceptable scientific standard, however I have significant reservations, as outlined above.

## References

[1] : Organizational performance and competitive advantage in SMEs: The role of green innovation and knowledge management. *Journal of Open Innovation: Technology, Market, and Complexity* .2025;11(2) : 10.1016/j.joitmc.2025.100532 10.1016/j.joitmc.2025.100532

[2] : Understanding the nexus of green innovation and sustainable performance: evidence from the tourism industry in Indonesia. *Innovation & Management Review* .2026;23: 10.1108/INMR-12-2023-025349-65 10.1108/INMR-12-2023-0253

# Reviewer report: Reviewer response for version 1

**DOI:** 10.5256/f1000research.202442.r509920

**Published:** 2026-08-12

**Authors:** Anas Pattaray, Lalu Ratmaja, Endang Sri Wahyuni

**Affiliations:** 1 Tour and Travel Business Management Study Program, Politeknik Pariwisata Lombok, Central Lombok Regency, West Nusa Tenggara, Indonesia; 2 Tour and Travel Business Management, Politeknik Pariwisata Lombok, Central Lombok Regency, West Nusa Tenggara, Indonesia

The article has a novelty value because it maps the development of research on strategic innovations in small and medium industry clusters (SMI) that are associated with regional tourism competitiveness through a bibliometric approach. The research identifies publication trends, collaboration networks, dominant themes, and shifts in innovation paradigms towards digitalization, smart tourism, and sustainability. However, the scientific contribution is still a mapping of the literature and has not yet produced a conceptual model or a new theoretical proposition.

**Please strengthen it with a more specific explanation of the research gap**. The author needs to affirm the new contribution of the research compared to previous bibliometric studies, for example, the contribution to the development of strategic innovation theory or tourism-based SMI strengthening policies.

 The literature review is quite extensive and covers the concepts of strategic innovation, digitalization, sustainability, smart tourism, and cross-sector collaboration. The author uses previous literature to establish the research context and explain the importance of innovation for SMI and the tourism sector. The number of references is also quite large, namely 11,383 references in the research database. However, the relationship between the main theory and the bibliometric results has not been fully elaborated.

**It is necessary to add a discussion of stronger theoretical literature**, such as Resource-Based View (RBV), Dynamic Capability Theory, or industrial cluster theory, to explain the mechanism of how innovation increases competitiveness. The latest literature related to AI, digital platforms, and tourism innovation ecosystems can also be expanded.

 The research method is quite clear and systematic. The research used bibliometric analysis with the framework of PRISMA and PICOC, using the Scopus database and Web of Science. From the initial 3,295 documents, a selection process was carried out until 195 articles were obtained for analysis. The use of VOSviewer and Biblioshiny is appropriate for the analysis of publication networks, keywords, citations, and collaborations. However, the reasons for keyword selection and the limitations of inclusion can still be deepened.

**It is necessary to provide stronger justification regarding the selection of the database**, the year range of the research, and the Boolean search formula. The author also needs to explain the potential bias due to only including English and open access/hybrid gold articles.

 The results of the research are presented quite systematically through the analysis of publication developments, author networks, institutions, countries, and keyword analysis. The research shows an increase in publications with an annual growth of 15.86%, 195 articles from 125 publication sources, as well as a shift in themes towards smart tourism, green economy, and digital integration. The study's conclusions are consistent with the results that integration of public policies, cluster infrastructure, and digital support is required. However, some interpretations of the results are too descriptive and not critical enough.

**It is necessary to add a critical analysis of the findings, for example,** why certain themes are dominant, the factors that cause trend changes, and the practical implications of each cluster. The results of the visualization should be more strongly associated with the theory used.

 The article has conveyed the implication that the development of tourism competitiveness requires regional policies that integrate the strengthening of cluster infrastructure and digital support. These implications are in accordance with the results of research that show the importance of digitalization, collaboration, and sustainability.

**The implications need to be made more specific for three groups of users**: (1) local governments in policy formulation, (2) SMI actors in business innovation strategies, and (3) academics for the next research agenda. The author also needs to explain the limitations of the research and opportunities for further research.

 The structure of the article has followed a clear scientific pattern (abstract, introduction, methods, results and discussion, conclusion). Table presentation and visualization help readers understand bibliometric results. However, there are some academic English issues, such as sentence repetition, inconsistent use of terms, and some grammatical errors. For example, the introduction has several repetitions of the idea of the importance of strategic innovation and digitalization.

**Articles need to go through academic proofreading**. Some paragraphs need to be summarized to be more effective. The author also needs to improve the transitions between sections so that the relationship between background, methods, results, and implications is stronger.

**Recommendation: **Major Revision. The article has the potential to be indexed after strengthening the research gap, deepening theory, improving the interpretation of results, and editing academic language.

Is the work clearly and accurately presented and does it cite the current literature?

Partly

If applicable, is the statistical analysis and its interpretation appropriate?

Partly

Are all the source data underlying the results available to ensure full reproducibility?

Partly

Is the study design appropriate and is the work technically sound?

Partly

Are the conclusions drawn adequately supported by the results?

Partly

Are sufficient details of methods and analysis provided to allow replication by others?

Partly

Reviewer Expertise:

• Sustainable Tourism Development • Halal and Muslim-Friendly Tourism • Destination Planning and Governance • Community-Based and Rural Tourism • Tourism and Cultural Heritage • Disability and Accessible Tourism • Tourism Human Resource Development

We confirm that we have read this submission and believe that we have an appropriate level of expertise to confirm that it is of an acceptable scientific standard, however we have significant reservations, as outlined above.

# Reviewer report: Reviewer response for version 1

**DOI:** 10.5256/f1000research.202442.r501503

**Published:** 2026-08-06

**Authors:** Shekhar Shekhar

**Affiliations:** 1 Jindal Global University, Sonipat, Haryana, India

I am sharing section-wise comments for improvement.

Abstract

  

1. The objective initially states that the study evaluates the evolution of strategic innovation trends within SMEs…and concludes with enhancing regional tourism competitiveness and long-term economic sustainability. The main objective should be enhancing tourism competitiveness and then state we are doing it through studying the evolution of innovation trends, etc. Synthesizing should not be the objective. The objective is boosting competitiveness through understanding innovation trends.
2. Bibliometric mapping is a systematic process. No need to use the word Rigorous.
3. In methods, instead of mentioning more on exclusion/inclusion criteria, give details on the analysis performed.
4. No need for author names and growth trajectory in the findings. Focus on what cluster components were found and how it is relevant for the objective of tourism competitiveness.

Introduction

  

1. It starts with how SMEs are the backbone of the Indonesian economy and then, in the third line, it repeats “Small and Medium Industries (SMIs) serve as the backbone of the local economy across various regions in Indonesia..” These are the same sentences.
2. The first two paragraphs are mixed up. Instead, I suggest that you devote the introductory para to SMEs and tourism competitiveness. Then the second paragraph to strategic innovation. Use a bridging sentence between the first two paragraphs.
3. This line “Comprehensive review by,7…” is not the correct way to have citations. Instead, write an argument and follow it up with a citation.
4. There is a lack of coherence in the introduction section. Focus more on problematization and then on the interconnection between tourism competitiveness and strategic innovation.
5. I suggest defining competitiveness first in the introduction for the readers. How are you stating it, in terms of pricing, attractiveness, or overall? Use a standard definition.

Methods

1. How did you arrive at these keywords only? Why only this timeline? Why only Scopus? Provide justification for all.
2. Why only VOSviewer and Bibliometrix, as others too can provide answers to key themes and questions?
3. In Figure 1, the right-side box stating exclusion/inclusion criteria, there is a non-English word.
4. Research Questions should have come after the introduction and been narrowed down through LR. Are these questions unique and not studied in the existing literature at all?

Results and Discussion

1. Figures 3, 4, and 5 are not self-explanatory. Try re-running the analysis and changing the parameter values to modify orientation. These are too cluttered.
2. The study starts in the introduction with Indonesia, but the analysis has been carried out in general. Non-Indonesian studies are also incorporated. Is the objective to synthesize the overall literature and then try to apply the findings to Indonesian SMEs? If so, there is a need to modify the introduction a lot more.
3. My question is, can the findings be generalized? Innovation in the context of China or Western nations may be different than in the Indonesian context.
4. Figure 11 is not at all readable. Similarly, the previous figures 9 and 10 add limited value in the context of the study’s objectives.

Overall Comment

1. The study ends with completing the bibliometric analysis. Ideally, it should have ended with developing the competitiveness of tourism SMEs in Indonesia.

Is the work clearly and accurately presented and does it cite the current literature?

Partly

If applicable, is the statistical analysis and its interpretation appropriate?

No

Are all the source data underlying the results available to ensure full reproducibility?

No source data required

Is the study design appropriate and is the work technically sound?

Yes

Are the conclusions drawn adequately supported by the results?

No

Are sufficient details of methods and analysis provided to allow replication by others?

No

Reviewer Expertise:

Economics, Business

I confirm that I have read this submission and believe that I have an appropriate level of expertise to state that I do not consider it to be of an acceptable scientific standard, for reasons outlined above.
